# Mortality and disability-adjusted life years in North Africa and Middle East attributed to kidney dysfunction: a systematic analysis for the Global Burden of Disease Study 2019

**DOI:** 10.1093/ckj/sfad279

**Published:** 2023-11-17

**Authors:** Mohammad-Mahdi Rashidi, Sahar Saeedi Moghaddam, Sina Azadnajafabad, Esmaeil Mohammadi, Amirmohammad Khalaji, Mohammad-Reza Malekpour, Mohammad Keykhaei, Negar Rezaei, Zahra Esfahani, Nazila Rezaei, GBD 2019 NAME Kidney Dysfunction Risk Factor Collaborators, Mohammad-Mahdi Rashidi, Sahar Saeedi Moghaddam, Sina Azadnajafabad, Esmaeil Mohammadi, Amirmohammad Khalaji, Mohammad-Reza Malekpour, Mohammad Keykhaei, Negar Rezaei, Zahra Esfahani, Nazila Rezaei, Mohsen Abbasi-Kangevari, Zeinab Abbasi-Kangevari, Samar Abd ElHafeez, Sherief Abd-Elsalam, Meriem Abdoun, Eman Abu-Gharbieh, Aqeel Ahmad, Ayman Ahmed, Sayer Al-Azzam, Rajaa M Al-Raddadi, Ala'a B Al-Tammemi, Javad Aminian Dehkordi, Mehrdad Amir-Behghadami, Jalal Arabloo, Mohammad Athar, Seyyed Shamsadin Athari, Mahsa Babaei, Hassan Babamohamadi, Nayereh Baghcheghi, Sara Bagherieh, Hamid Reza Baradaran, Akshaya Srikanth Bhagavathula, Vijayalakshmi S Bhojaraja, Milad Bonakdar Hashemi, Luciana Aparecida Campos, Azizallah Dehghan, Muhammed Elhadi, Waseem El-Huneidi, Ali Fatehizadeh, Alireza Feizkhah, Fataneh Ghadirian, Ali Gholami, Samer Hamidi, Hadi Hassankhani, Mahsa Heidari-Foroozan, Kamran Hessami, Kamal Hezam, Mohammad-Salar Hosseini, Soodabeh Hoveidamanesh, Rana Irilouzadian, Haitham Jahrami, Tannaz Jamialahmadi, Sathish Kumar Jayapal, Reema A Karasneh, Moien A B Khan, Yusra H Khan, Moawiah Mohammad Khatatbeh, Farzad Kompani, Hamid Reza Koohestani, Mohammed Kuddus, Soleiman Mahjoub, Ata Mahmoodpoor, Elaheh Malakan Rad, Ahmad Azam Malik, Tauqeer Hussain Mallhi, Mohammadreza Mobayen, Faezeh Mohammadi, Syam Mohan, Sara Momtazmanesh, Zuhair S Natto, Seyed Aria Nejadghaderi, Hassan Okati-Aliabad, Sina Rashedi, Amirfarzan Rashidi, Mahsa Rashidi, Elrashdy Moustafa Mohamed Redwan, Aly M A Saad, Fatemeh Saheb Sharif-Askari, Amirhossein Sahebkar, Morteza Saki, Abdallah M Samy, Elaheh Shaker, Jeevan K Shetty, Seyed Afshin Shorofi, Mohammad Tabish, Amir Taherkhani, Muhammad Umair, Seyed Mohammad Vahabi, Fereshteh Yazdanpanah, Arzu Yigit, Mazyar Zahir, Moein Zangiabadian, Ali H Mokdad, Christopher J L Murray, Mohsen Naghavi, Bagher Larijani, Farshad Farzadfar, Ali H Mokdad, Christopher J L Murray, Mohsen Naghavi, Bagher Larijani, Farshad Farzadfar

**Affiliations:** Non-Communicable Diseases Research Center, Endocrinology and Metabolism Population Sciences Institute, Tehran University of Medical Sciences, Tehran, Iran; Social Determinants of Health Research Center, Shahid Beheshti University of Medical Sciences, Tehran, Iran; Non-Communicable Diseases Research Center, Endocrinology and Metabolism Population Sciences Institute, Tehran University of Medical Sciences, Tehran, Iran; Kiel Institute for the World Economy, Kiel, Germany; Non-Communicable Diseases Research Center, Endocrinology and Metabolism Population Sciences Institute, Tehran University of Medical Sciences, Tehran, Iran; Non-Communicable Diseases Research Center, Endocrinology and Metabolism Population Sciences Institute, Tehran University of Medical Sciences, Tehran, Iran; School of Medicine, Tehran University of Medical Sciences, Tehran, Iran; Non-Communicable Diseases Research Center, Endocrinology and Metabolism Population Sciences Institute, Tehran University of Medical Sciences, Tehran, Iran; School of Medicine, Tehran University of Medical Sciences, Tehran, Iran; Non-Communicable Diseases Research Center, Endocrinology and Metabolism Population Sciences Institute, Tehran University of Medical Sciences, Tehran, Iran; Non-Communicable Diseases Research Center, Endocrinology and Metabolism Population Sciences Institute, Tehran University of Medical Sciences, Tehran, Iran; Students’ Scientific Research Center (SSRC), Tehran University of Medical Sciences, Tehran, Iran; Non-Communicable Diseases Research Center, Endocrinology and Metabolism Population Sciences Institute, Tehran University of Medical Sciences, Tehran, Iran; Endocrinology and Metabolism Research Center, Endocrinology and Metabolism Clinical Sciences Institute, Tehran University of Medical Sciences, Tehran, Iran; Non-Communicable Diseases Research Center, Endocrinology and Metabolism Population Sciences Institute, Tehran University of Medical Sciences, Tehran, Iran; Non-Communicable Diseases Research Center, Endocrinology and Metabolism Population Sciences Institute, Tehran University of Medical Sciences, Tehran, Iran; Non-Communicable Diseases Research Center, Endocrinology and Metabolism Population Sciences Institute, Tehran University of Medical Sciences, Tehran, Iran; Social Determinants of Health Research Center, Shahid Beheshti University of Medical Sciences, Tehran, Iran; Non-Communicable Diseases Research Center, Endocrinology and Metabolism Population Sciences Institute, Tehran University of Medical Sciences, Tehran, Iran; Non-Communicable Diseases Research Center, Endocrinology and Metabolism Population Sciences Institute, Tehran University of Medical Sciences, Tehran, Iran; Non-Communicable Diseases Research Center, Endocrinology and Metabolism Population Sciences Institute, Tehran University of Medical Sciences, Tehran, Iran; Kiel Institute for the World Economy, Kiel, Germany; Non-Communicable Diseases Research Center, Endocrinology and Metabolism Population Sciences Institute, Tehran University of Medical Sciences, Tehran, Iran; Kiel Institute for the World Economy, Kiel, Germany; Non-Communicable Diseases Research Center, Endocrinology and Metabolism Population Sciences Institute, Tehran University of Medical Sciences, Tehran, Iran; Non-Communicable Diseases Research Center, Endocrinology and Metabolism Population Sciences Institute, Tehran University of Medical Sciences, Tehran, Iran; School of Medicine, Tehran University of Medical Sciences, Tehran, Iran; Non-Communicable Diseases Research Center, Endocrinology and Metabolism Population Sciences Institute, Tehran University of Medical Sciences, Tehran, Iran; Students’ Scientific Research Center (SSRC), Tehran University of Medical Sciences, Tehran, Iran; Non-Communicable Diseases Research Center, Endocrinology and Metabolism Population Sciences Institute, Tehran University of Medical Sciences, Tehran, Iran; Non-Communicable Diseases Research Center, Endocrinology and Metabolism Population Sciences Institute, Tehran University of Medical Sciences, Tehran, Iran; Non-Communicable Diseases Research Center, Endocrinology and Metabolism Population Sciences Institute, Tehran University of Medical Sciences, Tehran, Iran; Social Determinants of Health Research Center, Shahid Beheshti University of Medical Sciences, Tehran, Iran; Non-Communicable Diseases Research Center, Endocrinology and Metabolism Population Sciences Institute, Tehran University of Medical Sciences, Tehran, Iran; Endocrinology and Metabolism Research Center, Endocrinology and Metabolism Clinical Sciences Institute, Tehran University of Medical Sciences, Tehran, Iran; Institute for Health Metrics and Evaluation, University of Washington, Seattle, WA, USA; Department of Health Metrics Sciences, School of Medicine, University of Washington, Seattle, WA, USA; Clinical Sciences Department, University of Sharjah, Sharjah, United Arab Emirates; Department of Medical Biochemistry, Shaqra University, Shaqra, Saudi Arabia; Institute of Endemic Diseases, University of Khartoum, Khartoum, Sudan; Swiss Tropical and Public Health Institute, University of Basel, Basel, Switzerland; Department of Clinical Pharmacy, Jordan University of Science and Technology, Irbid, Jordan; Department of Community Medicine, King Abdulaziz University, Jeddah, Saudi Arabia; Migration Health Division, International Organization for Migration, Amman, Jordan; Department of Family and Occupational Medicine, University of Debrecen, Debrecen, Hungary; Department of Applied Science and Technology, University of California Berkeley, Berkeley, CA, USA; Chemical Engineering Department, Tarbiat Modares University, Tehran, Iran; Road Traffic Injury Research Center, Tabriz University of Medical Sciences, Tabriz, Iran; Department of Health Service Management, Iranian Center of Excellence in Health Management, Tabriz, Iran; Health Management and Economics Research Center, Iran University of Medical Sciences, Tehran, Iran; Department of Medical Genetics, Umm Al-Qura University, Makkah, Saudi Arabia; Science and Technology Unit, Umm Al-Qura University, Makkah, Saudi Arabia; Department of Immunology, Zanjan University of Medical Sciences, Zanjan, Iran; Neuroscience Institute, Tehran University of Medical Sciences, Tehran, Iran; Department of Medicine, Stanford University, Palo Alto, CA, USA; Department of Nursing, Semnan University of Medical Sciences and Health Services, Semnan, Iran; Department of Nursing, Saveh University of Medical Sciences, Saveh, Iran; School of Medicine, Isfahan University of Medical Sciences, Isfahan, Iran; Department of Epidemiology, Iran University of Medical Sciences, Tehran, Iran; Ageing Clinical and Experimental Research (ACER), University of Aberdeen, Aberdeen, UK; Department of Health, Human Performance and Recreation, University of Arkansas, Fayetteville, AR, USA; Department of Anatomy, Royal College of Surgeons in Ireland Medical University of Bahrain, Busaiteen, Bahrain; Urology Department, Shahid Beheshti University of Medical Sciences, Tehran, Iran; Center of Innovation, Technology and Education (CITE), Anhembi Morumbi University, Sao Jose dos Campos, Brazil; College of Health Sciences, Abu Dhabi University, Abu Dhabi, United Arab Emirates; Department of Epidemiology and Community Medicine, Non-Communicable Diseases Research Center (NCDRC), Fasa, Iran; Faculty of Medicine, University of Tripoli, Tripoli, Libya; Department of Basic Medical Sciences, University of Sharjah, Sharjah, United Arab Emirates; Department of Environmental Health Engineering, Isfahan University of Medical Sciences, Isfahan, Iran; Department of Social Medicine and Epidemiology, Guilan University of Medical Sciences, Rasht, Iran; Psychiatric Nursing and Management Department, Shahid Beheshti University of Medical Sciences, Tehran, Iran; Department of Epidemiology and Biostatistics, Neyshabur University of Medical Sciences, Neyshabur, Iran; Non-Communicable Diseases Research Center, Neyshabur University of Medical Sciences, Neyshabur, Iran; School of Health and Environmental Studies, Hamdan Bin Mohammed Smart University, Dubai, United Arab Emirates; School of Nursing and Midwifery, Tabriz University of Medical Sciences, Tabriz, Iran; Independent Consultant, Tabriz, Iran; Non-Communicable Diseases Research Center, Endocrinology and Metabolism Population Sciences Institute, Tehran University of Medical Sciences, Tehran, Iran; School of Medicine, Shahid Beheshti University of Medical Sciences, Tehran, Iran; Maternal Fetal Care Center, Harvard University, Boston, MA, USA; Maternal Fetal Medicine Research Center, Shiraz University of Medical Sciences, Shiraz, Iran; Department of Applied Microbiology, Taiz University, Taiz, Yemen; Department of Microbiology, Nankai University, Tianjin, China; Student Research Committee, Tabriz University of Medical Sciences, Tabriz, Iran; Burn Research Center, Shahid Motahari Hospital, Tehran, Iran; Burn Research Center, Iran University of Medical Sciences, Tehran, Iran; School of Medicine, Shahid Beheshti University of Medical Sciences, Tehran, Iran; College of Medicine and Medical Sciences, Arabian Gulf University, Manama, Bahrain; Ministry of Health, Ministry of Health, Manama, Bahrain; Department of Nutrition, Mashhad University of Medical Sciences, Mashhad, Iran; Department of Food Science and Technology, Islamic Azad University, Quchan, Iran; Centre of Studies and Research, Ministry of Health, Muscat, Oman; Department of Basic Medical Sciences, Yarmouk University, Irbid, Jordan; Family Medicine Department, United Arab Emirates University, Al Ain, United Arab Emirates; Primary Care Department, NHS North West London, London, UK; Department of Clinical Pharmacy, Jouf University, Sakaka, Saudi Arabia; Department of Basic Medical Sciences, Yarmouk University, Irbid, Jordan; Children's Medical Center, Tehran University of Medical Sciences, Tehran, Iran; Social Determinants of Health Research Center, Saveh University of Medical Sciences, Saveh, Iran; Department of Biochemistry, University of Hail, Hail, Saudi Arabia; Cellular and Molecular Biology Research Center, Babol University of Medical Sciences, Babol, Iran; Department of Clinical Biochemistry, Babol University of Medical Sciences, Babol, Iran; Department of Anesthesiology and Critical Care, Tabriz University of Medical Sciences, Tabriz, Iran; Department of Pediatric Cardiology, Tehran University of Medical Sciences, Tehran, Iran; Rabigh Faculty of Medicine, King Abdulaziz University, Jeddah, Saudi Arabia; University Institute of Public Health, The University of Lahore, Lahore, Pakistan; Department of Clinical Pharmacy, Jouf University, Sakaka, Saudi Arabia; Burn and Regenerative Medicine Research Center, Guilan University of Medical Sciences, Rasht, Iran; School of Medicine, Iran University of Medical Sciences, Tehran, Iran; Substance Abuse and Toxicology Research Center, Jazan University, Jazan, Saudi Arabia; Center for Transdisciplinary Research, Saveetha Institute of Medical and Technical Science, Chennai, India; Non-Communicable Diseases Research Center, Endocrinology and Metabolism Population Sciences Institute, Tehran University of Medical Sciences, Tehran, Iran; Kiel Institute for the World Economy, Kiel, Germany; Department of Dental Public Health, King Abdulaziz University, Jeddah, Saudi Arabia; Department of Health Policy and Oral Epidemiology, Harvard University, Boston, MA, USA; School of Medicine, Shahid Beheshti University of Medical Sciences, Tehran, Iran; Non-Communicable Diseases Research Center, Endocrinology and Metabolism Population Sciences Institute, Tehran University of Medical Sciences, Tehran, Iran; Health Promotion Research Center, Zahedan University of Medical Sciences, Zahedan, Iran; Department of Cardiology, Tehran University of Medical Sciences, Tehran, Iran; Non-Communicable Diseases Research Center, Endocrinology and Metabolism Population Sciences Institute, Tehran University of Medical Sciences, Tehran, Iran; Rafsanjan University of Medical Sciences, Rafsanjan University of Medical Sciences, Rafsanjan, Iran; Department of Clinical Science, Islamic Azad University, Garmsar, Iran; Department Biological Sciences, King Abdulaziz University, Jeddah, Egypt; Department of Protein Research, Research and Academic Institution, Alexandria, Egypt; Cardiovascular Department, Zagazig University–Egypt, Zagazig, Egypt; Sharjah Institute of Medical Sciences, Sharjah, United Arab Emirates; Department of Applied Biomedical Research Center, Mashhad University of Medical Sciences, Mashhad, Iran; Biotechnology Research Center, Mashhad University of Medical Sciences, Mashhad, Iran; Department of Microbiology, Ahvaz Jundishapur University of Medical Sciences, Ahvaz, Iran; Department of Entomology, Ain Shams University, Cairo, Egypt; Non-Communicable Diseases Research Center, Endocrinology and Metabolism Population Sciences Institute, Tehran University of Medical Sciences, Tehran, Iran; Kiel Institute for the World Economy, Kiel, Germany; Department of Biochemistry, Royal College of Surgeons in Ireland Medical University of Bahrain, Busaiteen, Bahrain; Department of Medical-Surgical Nursing, Mazandaran University of Medical Sciences, Sari, Iran; Department of Nursing and Health Sciences, Flinders University, Adelaide, SA, Australia; Department of Pharmacology, Shaqra University, Shaqra, Saudi Arabia; Research Center for Molecular Medicine, Hamadan University of Medical Sciences, Hamadan, Iran; Medical Genomics Research Department, King Abdullah International Medical Research Center, Riyadh, Saudi Arabia; Department of Life Sciences, University of Management and Technology, Lahore, Pakistan; Faculty Of Medicine, Tehran University of Medical Sciences, Tehran, Iran; Department of Pediatric Allergy and Immunology, Tabriz University of Medical Sciences, Tabriz, Iran; Department of Pediatric Allergy and Immunology, Tehran University of Medical Sciences, Tehran, Iran; Department of Health Management, Süleyman Demirel Üniversitesi (Süleyman Demirel University), Isparta, Türkiye; Urology and Nephrology Research Center, Shahid Beheshti University of Medical Sciences, Tehran, Iran; School of Medicine, Shahid Beheshti University of Medical Sciences, Tehran, Iran; Institute for Health Metrics and Evaluation, University of Washington, Seattle, WA, USA; Department of Health Metrics Sciences, School of Medicine, University of Washington, Seattle, WA, USA; Institute for Health Metrics and Evaluation, University of Washington, Seattle, WA, USA; Department of Health Metrics Sciences, School of Medicine, University of Washington, Seattle, WA, USA; Institute for Health Metrics and Evaluation, University of Washington, Seattle, WA, USA; Department of Health Metrics Sciences, School of Medicine, University of Washington, Seattle, WA, USA; Students’ Scientific Research Center (SSRC), Tehran University of Medical Sciences, Tehran, Iran; Non-Communicable Diseases Research Center, Endocrinology and Metabolism Population Sciences Institute, Tehran University of Medical Sciences, Tehran, Iran; Students’ Scientific Research Center (SSRC), Tehran University of Medical Sciences, Tehran, Iran; Institute for Health Metrics and Evaluation, University of Washington, Seattle, WA, USA; Department of Health Metrics Sciences, School of Medicine, University of Washington, Seattle, WA, USA; Institute for Health Metrics and Evaluation, University of Washington, Seattle, WA, USA; Department of Health Metrics Sciences, School of Medicine, University of Washington, Seattle, WA, USA; Institute for Health Metrics and Evaluation, University of Washington, Seattle, WA, USA; Department of Health Metrics Sciences, School of Medicine, University of Washington, Seattle, WA, USA; Endocrinology and Metabolism Research Center, Endocrinology and Metabolism Clinical Sciences Institute, Tehran University of Medical Sciences, Tehran, Iran; Non-Communicable Diseases Research Center, Endocrinology and Metabolism Population Sciences Institute, Tehran University of Medical Sciences, Tehran, Iran; Endocrinology and Metabolism Research Center, Endocrinology and Metabolism Clinical Sciences Institute, Tehran University of Medical Sciences, Tehran, Iran

**Keywords:** cardiovascular diseases, chronic kidney disease, global burden of disease, kidney dysfunction, mortality

## Abstract

**Background:**

The study aimed to estimate the attributable burden to kidney dysfunction as a metabolic risk factor in the North Africa and Middle East (NAME) region and its 21 countries in 1990–2019.

**Methods:**

The data used in this study were obtained from the Global Burden of Diseases (GBD) 2019 study, which provided estimated measures of deaths, disability-adjusted life years (DALYs), and other epidemiological indicators of burden. To provide a better insight into the differences in the level of social, cultural, and economic factors, the Socio-Demographic Index (SDI) was used.

**Results:**

In the NAME region in 2019, the number of deaths attributed to kidney dysfunction was 296 632 (95% uncertainty interval: 249 965–343 962), which was about 2.5 times higher than in the year 1990. Afghanistan, Egypt, and Saudi Arabia had the highest, and Kuwait, Turkey, and Iran (Islamic Republic of) had the lowest age-standardized rate of DALYs attributed to kidney dysfunction in the region in 2019. Kidney dysfunction was accounted as a risk factor for ischemic heart disease, chronic kidney disease, stroke, and peripheral artery disease with 150 471, 111 812, 34 068, and 281 attributable deaths, respectively, in 2019 in the region. In 2019, both low-SDI and high-SDI countries in the region experienced higher burdens associated with kidney dysfunction compared to other countries.

**Conclusions:**

Kidney dysfunction increases the risk of cardiovascular diseases burden and accounted for more deaths attributable to cardiovascular diseases than chronic kidney disease in the region in 2019. Hence, policymakers in the NAME region should prioritize kidney disease prevention and control, recognizing that neglecting its impact on other diseases is a key limitation in its management.

KEY LEARNING POINTS **What was known**:

- Prior to this study, it was understood that kidney dysfunction contributes significantly to the global burden of non-communicable diseases, with cardiovascular diseases (CVDs) and gout being notable associated conditions.
- Disparities in the burden of kidney dysfunction were known to exist between regions, necessitating region-specific burden assessments.
- The study was necessary to quantify the burden of kidney dysfunction as a risk factor, rather than just a direct cause of disease, and to understand its impact in the specific context of the North Africa and Middle East (NAME) region.

**This study adds**:

- The study revealed a 2.5-fold increase in deaths attributed to kidney dysfunction in the NAME region from 1990 to 2019, with a stable age-standardized rate, indicating a significant rise in absolute numbers but not in relative terms.
- It was found that kidney dysfunction is a more significant risk factor for CVDs than for chronic kidney disease itself in terms of attributable deaths in the NAME region.
- The analysis indicates that the burden of kidney dysfunction in the NAME region is not uniformly distributed, with countries like Afghanistan, Egypt, and Saudi Arabia experiencing the highest rates, and Kuwait, Turkey, and Iran the lowest.

**Potential impact**:

- The findings may prompt healthcare policymakers in the NAME region to allocate more resources towards the prevention and control of kidney disease to mitigate its broader impact on health.
- This study could lead to enhanced surveillance and management strategies for kidney dysfunction, potentially improving outcomes for related conditions like CVDs.
- The evidence provided could support the development of targeted public health interventions and the optimization of healthcare delivery systems in the NAME region.

## INTRODUCTION

Non-communicable diseases (NCDs) had an emerging progressive contribution to the burden of diseases in the previous 30 years [1]. Therefore, prevention of diseases through controlling and decreasing exposure to related risk factors can effectively and drastically reduce the burden of diseases. Metabolic risk factors play one of the most important roles in this upsurge [2]. Kidney dysfunction, as one of these metabolic risk factors, contributes to increase the global burden of NCDs. Kidney dysfunction, in addition to its direct burden of kidney diseases, increases the premature deaths and burden of other diseases, such as cardiovascular diseases (CVDs) and gout [3, 4]. In 2015, the United Nations developed the sustainable development goals (SDGs) and target 3.4 calling on all countries to aim to reduce the national burden of premature deaths from NCDs by one-third through prevention and treatment by the year 2030 [5, 6]. To achieve target 3.4, prevention and control of kidney diseases had two-folded attainment because kidney diseases are the direct causes of death and on one hand, some premature deaths of other diseases are attributable to kidney dysfunction [3].

Kidney disease is well-demonstrated as a disease with a high economic burden [7]. While most of the expenditures seem to be for end-stage kidney disease (ESKD) patients that need renal replacement therapies, such as dialysis and kidney transplantation, the evidence shows chronic kidney diseases (CKDs), especially in lower stages, have more economic burden due to their much higher prevalence [7]. However, there are large variations in CKD-related deaths between regions and countries [8]. Therefore, as an economic sight, both ESKD and CKD should be important for policymakers, and medical authorities should implement policies for the prevention of the early stages of CKD [9]. Reporting the burden of chronic kidney disease alone is insufficient to highlight the importance of kidney diseases; it is crucial to also report the burden and premature deaths resulting from kidney diseases through other associated conditions.

North Africa and Middle East (NAME) in the global burden of disease (GBD) 2019 study counts as one of the seven GBD super-regions [1]. Despite religious and cultural similarities among NAME countries, significant economic disparities exist between them. The socioeconomic disparity and heterogeneity among them have remained unchanged over the past 30 years [10]. Improvements in the availability and delivery of healthcare services in the countries of this region over the past decade have led to increased life expectancy and a higher proportion of elderly individuals in the population [11–13]. This is making aging an alarming trend in the region, which is also projected to intensify in the future [11]. The GBD 2017 study found that impaired kidney function had a high global burden, with 772.0 disability-adjusted life years (DALYs) per 100 000 population and ranked 16th among 84 risk factors [14]. However, in the NAME region, the burden was even higher with 1093.8 DALYs per 100 000 and ranked 10th among 84 risk factors [14]. Therefore, accurate monitoring of the current situation and prioritization of preventive strategies for kidney diseases at the regional and national levels is urgently needed in NAME.

In a previous publication, a group of authors from this region tried to evaluate the burden of CKD in the NAME region [15]. The mentioned publication focuses on chronic kidney disease as a cause of disease under the category of non-communicable diseases in the GBD hierarchy [15]. In contrast, our current study examines the burden attributable to kidney dysfunction as a risk factor for a variety of diseases, categorized under metabolic risks in the same GBD framework. This differentiation primarily stems from the GBD's methodological approach to burden estimation, which categorizes diseases and risk factors separately, based on a comprehensive literature review [1, 2]. Therefore, we believe that it is essential not only to study the burden of chronic kidney disease but also to explore the disease burden attributable to kidney dysfunction as a risk factor. This nuanced understanding can provide valuable insights for better disease management and can inform epidemiological measures. Therefore, this study aimed to estimate the burden attributable to kidney dysfunction as a metabolic risk factor in NAME and its 21 countries from 1990 to 2019. This study brings estimations from all available data about kidney dysfunction by the 2019 updates of GBD study.

## MATERIALS AND METHODS

### Data source overview

This study was a part of the GBD 2019 study, and it reports the attributable burden of kidney dysfunction as a risk factor. The GBD 2019 study is a systematic, scientific effort to estimate the burden of diseases and risk factors by age and sex across 204 countries and territories from 1990 to 2019 [1, 2]. The GBD study utilizes updated methods and data sources, including censuses, surveys, registries, and other sources, to provide a comprehensive understanding of diseases and risk factors in each country and territory [2]. The data were obtained through a systematic review of global sources, with further details provided in a separate discussion, elsewhere [2]. Exclusion criteria were non-representative population samples and studies not reporting CKD by stages. This study uses the estimation of the attributable burden of risk factors following the general framework that was set up to use comparative risk assessment (CRA) in GBD study since the year 2002 [16].

The CRA framework assesses two types of risk: attributable burden, which reduces current disease burden if past exposure to a risk factor had changed; and avoidable burden, which reduces potential future disease burden if current exposure to a risk factor changes [16]. The study applied standard epidemiological measures such as death rate and used calculated measures such as years of life lost (YLLs), years lived with disability (YLDs), and DALYs to demonstrate the burden or attributable burden of diseases and risk factors.

Updates of the GBD study consist of considering new diseases, new risk factors, new sources of data, and updates in methods of the study. In GBD 2019, kidney dysfunction was undertaken in a reassessment of the relationship of dose-response assumption and the previous assumptions that their risk curves were log-linear diminished. The GBD 2019 study was accomplished according to the Guidelines for Accurate and Transparent Health Estimates Reporting (GATHER) for reporting estimations [1]. More details about the study design, models, and calculations used by the GBD 2019 study to estimate the burden of diseases and attributable burden for risk factors were discussed comprehensively in previously published papers [1, 2].

Access to the results of the study is available on an online portal named GBD results tool [17]. The GBD 2019 study classified the NAME region as both a super-region and a region, comprising 21 countries: Afghanistan, Algeria, Bahrain, Egypt, Iran (Islamic Republic of), Iraq, Jordan, Kuwait, Lebanon, Libya, Morocco, Oman, Palestine, Qatar, Saudi Arabia, Sudan, Syrian Arab Republic, Tunisia, Turkey, United Arab Emirates, and Yemen. The GBD 2019 study uses hierarchical leveling to categorize causes. In the first level, causes are categorized into three categories: (i) communicable, maternal, neonatal, and nutritional diseases; (ii) non-communicable diseases; and (iii) injuries. Also, the GBD study uses a hierarchical leveling to categorize risk factors. In the first level, risk factors are categorized into three categories of behavioral, environmental/occupational, and metabolic risk factors. Kidney dysfunction is categorized in the second level and accounted as a subcategory of metabolic risk factors. The risk-outcome pairs were defined based on related previous qualified evidence.

### Study outcomes and statistical analysis

The burden attributed to kidney dysfunction was demonstrated by deaths, YLLs, YLDs, and DALYs and the exposure to risk factors was calculated through summary exposure value (SEV) [2]. To report attributable deaths, YLLs, YLDs, and DALYs the number and rate per 100 000 of these variables were used. The age-standardized rate was also reported to make a reliable comparison of the burden attributed to kidney dysfunction between different populations without the confounding effect of differences in the age structures of the populations. By applying the 2019 updated version of the world population age standard of the GBD study, age-standardized rates were estimated [1]. In the GBD 2019 study, the burden attributable to kidney dysfunction was estimated from early neonatal to the elderly. In this study, the age ranges of under 20, 20 to 54, 55 plus, and all ages were used for visualization because the previous study showed age groups included in these mentioned age groups have similarities and some common situations [3]. The SEV was reported on a scale from 0% to 100% on which an increase in SEV of a risk factor indicates the exposure to that risk factor increased and a decrease in SEV indicates the exposure was reduced [2]. To provide a better insight into the differences in the level of social, cultural, and economic factors among countries of the region, the SDI was used in this study. To calculate SDI, a composite average of the rankings of three indices of total fertility rate in those under 25 years old, mean education for those aged 15 years or older, and lag‐distributed income per capita were used, more information was provided elsewhere [1]. Kidney dysfunction was defined as estimated glomerular filtration rate (eGFR) less than 60 ml/min/1.73m^2^ or albumin to creatinine ratio (ACR) greater than or equal to 30 mg/g. In this study, kidney dysfunction was accounted as a risk factor for CVDs, CKD, and gout. Based on the previous studies, the theoretical minimum-risk exposure level (TMREL) of kidney dysfunction was used as a threshold at which increased cardiovascular and gout events occur secondary to kidney dysfunction [2]. The TMREL is ACR less than 30 mg/g and eGFR greater than or equal to 60 ml/min/1.73m^2^. From CVDs, subcategories of ischemic heart disease (IHD), stroke, and peripheral artery disease had burdens attributable to kidney dysfunction. Ischemic stroke and intracerebral hemorrhage are subcategories of stroke in the GBD causes hierarchy and some of their burdens were attributable to kidney dysfunction. Also, CKD is subcategorized into CKD due to diabetes mellitus type 1, CKD due to diabetes mellitus type 2, CKD due to hypertension, CKD due to glomerulonephritis, and CKD due to other and unspecified causes. In the GBD study, to consider the uncertainty of data sources, measurement error, and modellings, all the items were quantified 1000 times in the models. To produce the final estimates with 95% UIs, the 25th and 975th ordered values of 1000 draws of the posterior distribution were used [3]. Primary data management, statistical analysis, and visualization in this study were performed by R for Windows version 4.0.5 (The R Project for Statistical Computing, https://www.r-project.org/).

### Role of the funding source

The funders of the study had no role in study design, data collection, data analysis, data interpretation, or the writing of the report. The corresponding author had full access to the data in the study and final responsibility for the decision to submit for publication.

## RESULTS

### Deaths, DALYs, and SEV

In the year 2019 in NAME, the total number of deaths attributed to kidney dysfunction was 296 632 (95% UI: 249 965–343 962) which was about 2.5 times of the year 1990. Also, the total number of DALYs in 2019 was 7 184 796 (6 159 596–8 242 923) which was 2.2 times of the year 1990. In the year 2019 in the region, the attributed age-standardized rate of deaths was 83.4 (69.8–97.3) per 100 000 compared to 84.3 (71.1–99.1) in the year 1990. Also, the attributed age-standardized rate of DALYs in 2019 was 1691.0 (1449.4–1946.0) per 100 000, which in the year 1990 was 1767.1 (1532.2–2015.1) (Table 1). In 2019 in the region, the SEV of kidney dysfunction was 26.9 (20.4–34.6) for both sexes with an increase of 32.3% (23.1–44.0) compared to 1990. The SEV of kidney dysfunction in the countries of the region was almost similar and considering 95% UIs, no significant differences were seen between them (Table S1, see online supplementary material).

**Table 1:** The: North Africa and Middle East region trend of epidemiologic indices attributed to kidney dysfunction, attributed number for all ages and age-standardized rates, for males, females, and both sexes, in 1990 and 2019 and % of changes in the 1990–2019 period.

		Year								
		1990			2019			% Change (1990 to 2019)		
Measure	Age, Metric	Both	Female	Male	Both	Female	Male	Both	Female	Male
Deaths	Attributed all ages number	120 312 (103 802 to 138 364)	60 081 (51 762 to 70 255)	60 231 (51 000 to 69 955)	296 632 (249 965 to 343 962)	143 880 (121 226 to 167 839)	152 751 (128 538 to 179 204)	146.6 (117.1 to 173.5)	139.5 (106.2 to 169.1)	153.6 (121 to 186.3)
	Attributed age-standardized rate (per 100 000)	84.3 (71.1 to 99.1)	83.7 (70.4 to 99.2)	84.8 (70.6 to 100)	83.4 (69.8 to 97.3)	82.2 (68.3 to 96.6)	84.5 (70 to 99.9)	−1.1 (−13.7 to 9.4)	−1.8 (−16.3 to 9.9)	−0.3 (−13.7 to 12.1)
DALYs	Attributed all ages number	3 277 448 (2 896 209 to 3 677 544)	1 638 933 (1 457 390 to 1 832 925)	1 638 515 (1 423 771 to 1 866 793)	7 184 796 (6 159 596 to 8 242 923)	3 450 188 (2 965 498 to 3 970 895)	3 734 608 (3 138 504 to 4 352 083)	119.2 (99.3 to 142.9)	110.5 (87.6 to 135.5)	127.9 (104.5 to 156.2)
	Attributed age-standardized rate (per 100 000)	1767.1 (1532.2 to 2015.1)	1765.5 (1546.9 to 2011.4)	1765.9 (1510.2 to 2043.4)	1691 (1449.4 to 1946)	1658 (1426.3 to 1911.2)	1722.3 (1453.9 to 2006.2)	−4.3 (−14.9 to 5.2)	−6.1 (−18.1 to 4.9)	−2.5 (−13.8 to 8.9)
YLLs	Attributed all ages number	2 982 791 (2 630 219 to 3 360 891)	1 466 175 (1 301 761 to 1 645 589)	1 516 617 (1 315 443 to 1 726 494)	6 213 627 (5 238 636 to 7 226 519)	2 931 206 (2 485 128 to 3 416 417)	3 282 421 (2 752 361 to 3 863 653)	108.3 (86.5 to 133.6)	99.9 (75.4 to 127.7)	116.4 (91.3 to 146.1)
	Attributed age-standardized rate (per 100 000)	1628.6 (1413 to 1858.5)	1603.5 (1391.1 to 1853.1)	1650.7 (1404.1 to 1908.2)	1487.7 (1259.8 to 1721.5)	1439.5 (1217.1 to 1674.8)	1533.1 (1291.1 to 1795.1)	−8.7 (−19.6 to 1.8)	−10.2 (−22.6 to 1.6)	−7.1 (−18.9 to 5)
YLDs	Attributed all ages number	294 657 (216 296 to 387 305)	172 759 (126 947 to 226 028)	121 898 (88 363 to 160 440)	971 169 (707 482 to 1 267 687)	518 982 (377 778 to 669 683)	452 187 (327 945 to 597 557)	229.6 (212.9 to 247.9)	200.4 (185.6 to 217.4)	271 (249 to 292.3)
	Attributed age-standardized rate (per 100 000)	138.4 (102.4 to 179.7)	162 (120.9 to 210.6)	115.3 (83.6 to 151.7)	203.2 (149.5 to 264.3)	218.5 (162.1 to 282.3)	189.2 (136.9 to 252.8)	46.8 (40.8 to 53)	34.9 (29.3 to 40.9)	64.1 (56.4 to 72.1)

*Data in parentheses are 95% uncertainty intervals (95% UIs). The symbol − is used to denote that a number is negative and means decrease in % change. DALYs: disability-adjusted life years; YLLs: years of life lost; YLDs: years lived with disability.

In 2019, the age-standardized rate of DALYs attributed to kidney dysfunction for males was 1722.3 (1453.9–2006.2) and for females was 1658.0 (1426.3–1911.2) with no significant difference (Table 1). Also, we did not find significant differences between males and females in all countries of the region, except Qatar. In Qatar, the age-standardized DALY attributed to females was significantly more with a rate of 2450.9 (2009.1–2962.5) than males with a rate of 1521.3 (1186.8–1922.3) (Table S2, see online supplementary material).

**Figure 1:**
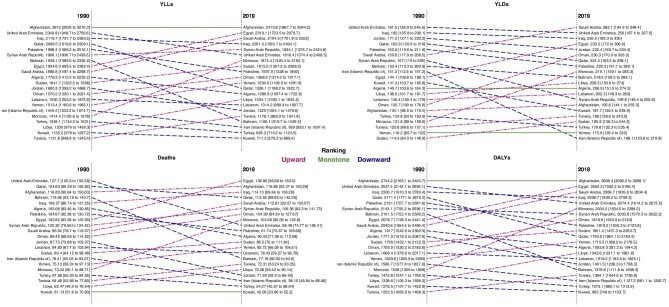
Attributed age-standardized rate of YLLs, YLDs, deaths, and DALYs of kidney dysfunction in both sexes in 21 countries of the region by ranking of each country in 1990 and 2019. DALYs: disability-adjusted life years; YLLs: years of life lost; YLDs: years lived with disability.

**Figure 2:**
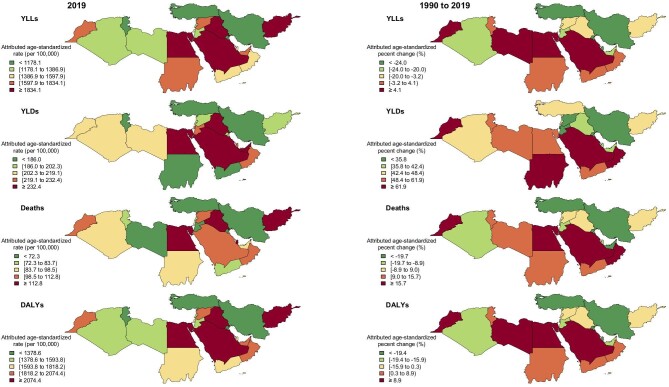
Attributed age-standardized rate of YLLs, YLDs, deaths, and DALYs of kidney dysfunction in both sexes, countries were stratified by colors for quintiles in 2019 (left side) and percentage change of these indices from 1990 to 2019 (right side). DALYs: disability-adjusted life years; YLLs: years of life lost; YLDs: years lived with disability.

Egypt, Afghanistan, and Iraq had the highest age-standardized deaths attributed to kidney dysfunction with rates of 128.7 (96.7–163.5), 118.9 (92.4–150.3), and 114.1 (89.6–136.3) per 100 000, respectively in 2019. However, Kuwait, Turkey, and Iran (Islamic Republic of) had the lowest age-standardized deaths attributed to kidney dysfunction with the rates of 42.1 (33.9–52.2), 54.3 (42.6–68), and 58.2 (48.8–68.1) per 100 000, respectively (Figs 1 and 2; Table S2, see online supplementary material).

### YLLs and YLDs

The attributed all ages’ number of YLLs was 6 213 627 (5 238 636–7 226 519) in NAME in 2019 and it was 2.1 times of the year 1990. The attributed age-standardized rate of YLLs was 1487.7 (1259.8–1721.5) per 100 000 in the region in 2019 but did not have a significant difference with the rate of the year 1990 that was 1628.6 (1413.0–1858.5) per 100 000 (Table 1). Afghanistan, Egypt, and Saudi Arabia had the highest age-standardized rates of YLLs attributed to kidney dysfunction and Kuwait, Turkey, and Iran (Islamic Republic of) had the lowest rates, respectively (Figs 1 and 2; Table S2, see online supplementary material).

Both attributed all ages’ number and attributed age-standardized rate of YLDs facing an increase in the region. The attributed all ages’ number of YLDs was 971 169 (707 482–1 267 687) in NAME in 2019 and it was 3.3 times of the year 1990. The attributed age-standardized rate of YLDs was 203.2 (149.5–264.3) per 100 000 in the region in 2019 which in 1990 was 138.4 (102.4–179.7) per 100 000 (Table 1). Saudi Arabia, United Arab Emirates, and Iraq had the highest age-standardized rates of YLDs attributed to kidney dysfunction and Iran (Islamic Republic of), Yemen, and Turkey had the lowest rates, in decreasing order (Figs 1 and 2; Table S2, see online supplementary material).

Most of the burden attributed to kidney dysfunction was related to YLLs rather than YLDs. But there were some exceptions in some age groups. In Qatar, in the under 20 and 20 to 54 age groups, the YLD attributed to kidney dysfunction was more than YLLs. Also, in the under-20 age group, in Kuwait, Oman, and Saudi Arabia the YLDs attributed to kidney dysfunction were more than YLLs. In the 55-plus age group, the burden related to YLLs was more than YLDs in all countries of the region with no exception (Fig. S1, see online supplementary material). Because of the much higher share of YLLs than YLDs in burden attributed to kidney dysfunction, the ranking of countries by DALYs is much similar to the ranking by YLLs (Fig. 1).

### Responsible causes for kidney dysfunction's attributable burden

**Figure 3:**
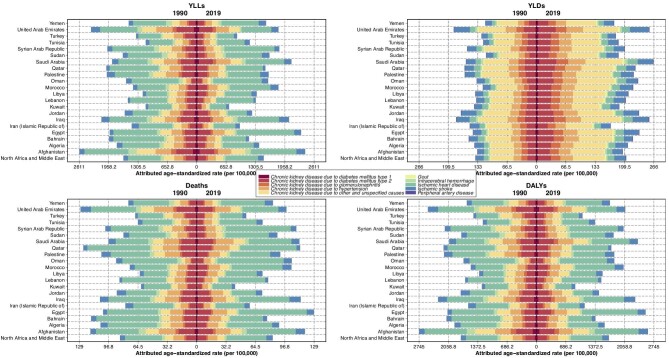
Attributed age-standardized rate of YLLs, YLDs, deaths, and DALYs of kidney dysfunction in both sexes due to causes in years 1990 and 2019. DALYs: disability-adjusted life years; YLLs: years of life lost; YLDs: years lived with disability.

In the GBD 2019 study, the burden attributable to kidney dysfunction was due to CKD, IHD, ischemic stroke, intracerebral hemorrhage, peripheral artery disease, and gout. Because CKD is a chronic situation of impaired function of the kidney, all of the CKD burdens is attributable to kidney dysfunction as a risk factor. In NAME, 16.4% (12.8–20.1) of all ages DALYs due to IHD was attributable to kidney dysfunction. Also, 26.0% (21.2–31.0), 18.8% (15.8–22.0), and 10.5% (8.7–12.3) of total all ages DALYs due to peripheral artery disease, gout, and stroke was attributable to kidney dysfunction in the region, respectively. Egypt, Syrian Arab Republic, and Oman due to IHD; Afghanistan, Saudi Arabia, and United Arab Emirates due to CKD; Iraq, Afghanistan, and Saudi Arabia due to stroke; Bahrain, Turkey, and Qatar due to peripheral artery disease; and Qatar, Saudi Arabia, and United Arab Emirates due to gout had the highest age-standardized rate of DALYs attributed to kidney dysfunction in the region in 2019. By level 4 or the most detailed level of causes hierarchy in every 21 countries of the region, IHD than other causes had the highest contribution in YLLs, deaths, and DALYs attributed to kidney dysfunction while in YLDs, CKD due to other and unspecified causes had the highest contribution (Fig. 3; Table S3, see online supplementary material).

### SDI

**Figure 4:**
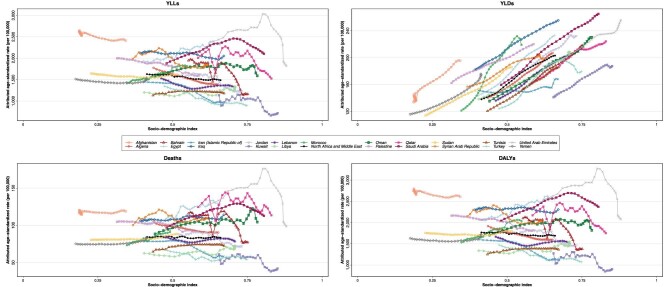
Attributed age-standardized rate of YLLs, YLDs, deaths, and DALYs of kidney dysfunction in both sexes by SDI of each country of the region and mean of the region from 1990 to 2019. DALYs: disability-adjusted life years; YLLs: years of life lost; SDI: socio-demographic index; YLDs: years lived with disability.

The countries in NAME have a wide diversity in SDIs ranging from 0.34 to 0.88 in 2019. Also, most countries of the region had significant growth in their SDI from 1990 to 2019. In the region and most countries of the region, despite the increase of SDIs, the attributed age-standardized rate of YLLs, deaths, and DALYs did not change significantly. More importantly, in most countries of the region, the attributed age-standardized rate of YLDs increased with the increase of SDI (Fig. 4). By grouping the countries of the region by SDI quintiles in 2019, four countries of the region with the highest age-standardized rate of DALYs, which were Afghanistan, Egypt, Saudi Arabia, and Iraq, were grouped as low SDI, low-middle SDI, high SDI, and middle SDI, respectively (Fig. S2, see online supplementary material). High SDI countries of the region, except for Kuwait, had higher age-standardized rates of DALYs and deaths attributable to kidney dysfunction than the mean of the region in 2019.

## DISCUSSION

In the year 2019, the number of deaths and DALYs attributable to kidney dysfunction in NAME increased to 2.5 and 2.2 times from 1990, respectively. The age-standardized rate of DALYs, deaths, and YLLs did not change from 1990 to 2019 in the region, while the age-standardized rate of YLDs increased by 47%. This study found that in NAME in 2019, IHD is the most responsible cause of deaths attributable to kidney dysfunction followed by CKD and stroke. As IHD and stroke are subcategories of CVDs, the attributed burden of kidney dysfunction due to CVDs was higher than CKDs. Four countries of the region with the highest age-standardized rate of DALYs attributable to kidney dysfunction in 2019 were Afghanistan, Egypt, Saudi Arabia, and Iraq. Overall, in our study low SDI and high SDI countries of the region had more burdens attributable to kidney dysfunction than other countries of the region in 2019.

Kidney dysfunction resulted in more attributable deaths than low physical activity, child and maternal malnutrition, occupational risks, or non-optimal temperature in 2019 [2]. Kidney dysfunction accounted for about 15% of total deaths attributable to all 87 risk factors defined by the GBD 2019 study in NAME while at global level it accounted for only 9% of attributable deaths [2, 17]. In our study regarding disability, considering YLDs, the highest attributable burden of kidney dysfunction was related to CKD. On the other hand, in terms of deaths, considering YLLs, the highest attributable burden of kidney dysfunction was related to CVDs. In addition to the fact that kidney dysfunction is a risk factor for the burden of CVDs, there is a bidirectional relationship, so that in cardiorenal syndrome types 1 and 2, acute and chronic heart failure lead to renal dysfunction, respectively [18]. The age-standardized rates of CVDs deaths including IHD and stroke had a declining trend in the region from 1990 to 2019 [17, 19, 20]. Such declining trends also will help the attributable burden of kidney dysfunction.

Although in our study the regional age-standardized DALY rate did not change through the previous 30 years, because of the reduction in total DALYs attributed to all risk factors, the share of kidney dysfunction from the burden of all risk factors increased significantly from 1990 to 2019 in the region. From all 21 GBD regions of the world that were defined in the GBD 2019 study, NAME had the highest age-standardized death rate attributed to kidney dysfunction. For the age-standardized DALY rate, NAME had the second rank in these 21 regions [17]. Also, we can see from the top 16 countries with the most attributed age-standardized death rate in 204 countries and territories, half of them were from the NAME region in the year 2019. These eight countries are Egypt, Afghanistan, Iraq, Qatar, Saudi Arabia, Syrian Arab Republic, Oman, and Morocco [17]. Some of these countries have been facing conflicts and wars while some others are high-income countries with stable economies [21, 22]. As we can see, the situation of kidney dysfunction in the NAME region is critical and prevention interventions in this region should be considered [17]. All prevention strategies should be implemented in health services. Health sectors should make sure to deliver the strategies in rural areas, which may experience substantial inequities [23, 24]; from general lifestyle modifications to specific recommendations in patients with kidney diseases. Some preventions such as screening at-risk patients, early therapy initiation and disease progression monitoring, aggressive blood pressure control, hyperglycemia proper management, smoking cessation, and adequate nutrition should be emphasized in the local health sectors [25, 26]. Recommending a diet that is gentle on the kidneys, such as a low-salt, low-fat, low-cholesterol, and high fruits and vegetables in the diet is helpful [27]. In the NAME region there are reports of widespread healthcare service limitations, such as dialysis shortages in local facilities [28]. In all these countries, even countries with low resources, using low-cost surveillance systems for kidney diseases could help to find the gaps and bridge them. Surveillance systems were used in other high- and also low-income countries and had valuable outcomes [29].

The regional age-standardized DALYs, deaths, and YLLs rates attributable to kidney dysfunction did not change significantly from 1990 to 2019, but the YLDs rate is facing growth. The growth of YLDs can be due to advances in the use of renal replacement therapies, especially hemodialysis, which save lives but come along with high levels of disabilities [30]. Dialysis in comparison with kidney transplantation imposes a much higher burden and increases the risk of premature death [31]. Also, in a study in Sweden, it was shown that kidney transplantation has considerable cost savings in comparison with dialysis for the healthcare system [32]. Therefore, prioritizing kidney transplantation and related legislations for long-term management of patients with CKD can reduce the growth in YLDs.

The NAME region consists of low- to high-income countries. Even now in some countries of the region, specialized medical services are not generally available while some others have better situations that can address the current medical standards [33–37]. As conflicts and wars change the focus of policy makers from chronic disease to acute ones and also bring destruction to facilities, countries experiencing conflicts face worsening of the situation in the burden of chronic disease that continues in the upcoming years after conflicts and wars [38]. In countries with the experience of conflict and war, international support and donation can significantly make the situation better and in countries with stable economies, considering more about kidney diseases and their attributed burden by related health-policy priorities can improve the situation. Also, inequalities in health service delivery are a major concern in countries of the region [39]. Afghanistan had the most age-standardized DALYs rate attributed to kidney dysfunction in 2019 in the region. Afghanistan as a country involved in long-term conflicts and has many problems in providing services and infrastructures [40]. According to the 2016 diabetes country profiles report of the World Health Organization (WHO), renal replacement therapy by dialysis or transplantation is not generally available in Afghanistan [41]. A study in 2016 reported that, for the 33 million people of Afghanistan there were 10 nephrologists and 200 hemodialysis machines, which were concentrated in major cities [42]. Therefore, lack of facilities and services in Afghanistan prevented improvement of the situation of kidney diseases. Similarly, some other countries of the region are facing or recently faced conflicts or wars that resulted in lack of infrastructures and services that consequently led to more deaths attributed to NCDs [35, 43–46]. Due to the complex and costly nature of kidney disease care, it is closely linked to the health policies implemented in each country and the resources allocated to healthcare. In particular, the lack of infrastructure affects more patients suffering from ESKD because they need renal replacement therapies to survive [47]. In the treatment of patients with high-risk CKD, access to essential drugs in addition to manpower and renal replacement therapy methods is very important in minimizing this risk factor. Not only people in Afghanistan and some other countries may have limited access to healthcare services, but also refugees from these countries suffer from lack of healthcare services access in their destination countries and try to meet their needs with the help of charities, international organizations, or social workers [48, 49]. Disparity in income, social, and cultural aspects of this region is high, which has been depicted by differences in countries’ SDI. Lack of infrastructures and services in low SDI countries reasonably leads to a higher burden of disease but also unexpectedly high SDI countries of the NAME region, except Kuwait, had higher attributable burden to kidney dysfunction than most other countries in 2019. Therefore, prioritizing kidney disease management is crucial in all countries of the NAME, as we can see it has been neglected in some high-income countries as well as low-income ones.

To our knowledge, this study was the first study focusing on kidney dysfunction as a risk to other diseases in the NAME region. Accounting burdens attributable to risks helps to have a better insight into prevention programs. Therefore, the findings of this study can be used by authorities and policymakers of countries of the region and also help advocates seeking better kidney disease management in their countries. This study also had some limitations. As mentioned before, the assumption of kidney dysfunction in the GBD 2019 study was undertaken as a reassessment of the relationship of dose-response and the effect of previous assumptions that their risk curves were log-linear were diminished [2]. However, this reassessment only applied on kidney dysfunction, dietary risks, and air pollution and other continuous risks in the GBD 2019 study were assumed on the log-linear relationship [2]. This difference in methods of assumptions could lead to some uncertainty about ranking of risks. In future studies, it is possible that the reassessment of the dose-response relationship applies on other risks, and it may lead to some differences in ranking of risk factors and may change some risk-outcomes. To find the attributed burden of each risk factor there was a need to consider the assessment of the joint effects of risk factors. GBD 2019 tried to use the best possible methods and modellings to report the most possible accurate results but because of the nature of assumption it can be with errors and uncertainty [2]. Because the GBD study collects data from various studies and other sources to form its estimations, a key limitation in assessing the attributable burden of risk factors and exposure measurements lies in the availability and quality of primary data. This is particularly true in countries with struggling healthcare systems, as well as those facing other economic and political challenges, which are numerous in the NAME region. Despite this limitation, the GBD study offers the most reliable estimates concerning the burden attributable to various risk factors, including kidney dysfunction, which is the focus of this study. These estimates are applicable across different geographical areas and can be used to improve public health, prevent further disease burden, and address disparities among populations.

## CONCLUSIONS

The study findings showed that although the total number of deaths and DALYs attributed to kidney dysfunction in the NAME region more than doubled between 1990 and 2019, the age-standardized rate of burden remained relatively stable during this period. However, among all 21 regions of the world, NAME had the highest age-standardized rate of death attributable to kidney dysfunction from 1990 to 2019. The number of CVDs deaths that were attributable to kidney dysfunction was more than the number of CKDs deaths in the region in 2019. Hence, it is crucial to avoid the pitfall of solely focusing on the direct burden of kidney diseases and overlooking the burden imposed by other diseases, particularly cardiovascular diseases, which are associated with kidney dysfunction. Given the critical situation in the study's region compared to other global regions, there is an urgent need to focus on kidney disease prevention and control. Improving data registries for kidney diseases, enhancing access to dialysis, and planning extensive research on the specific causes of kidney disorders in individual countries within the NAME region are essential steps to curb the burden attributable to this risk factor.

## Supplementary Material

sfad279_Supplemental_File

## Data Availability

The data generated and/or analysed in this study can be accessed through the GBD results tool portal at [https://vizhub.healthdata.org/gbd-results/].
