# Supplementary material for: Mortality and disability-adjusted life years in North Africa and Middle East attributed to kidney dysfunction: a systematic analysis for the Global Burden of Disease Study 2019
Source: Clin Kidney J. 2023 Nov 17;17(1):sfad279. doi: 10.1093/ckj/sfad279 (PMC10823484; doi:10.1093/ckj/sfad279)
Supplement: sfad279_Supplemental_File [file sfad279_supplemental_file.pdf]

Supplementary materials for “Kidney dysfunction’s attributed mortality and disability-adjusted life years in the North Africa and Middle East region, 1990-2019: a systematic analysis for the Global Burden of Disease Study 2019”

Supplementary Table 1. The SEV of North Africa and Middle East and 21 countries of the region in males, females, and both sexes in years 1990 and 2019 and percentage change of this two years..Page 2

Supplementary Table 2. The 21 countries of the region trend of epidemiologic indices attributed to kidney dysfunction, attributed number for all ages and age-standardized rates, for males, females, and both sexes, in 1990 and 2019 and percent of changes in the 1990-2019 period...Page 4

Supplementary Table 3. The causes trend of epidemiologic indices attributed to kidney dysfunction, attributed number for all ages and age-standardized rates, for males, females, and both sexes, in 1990 and 2019 and percent of changes in the 1990-2019 period..Page 19

Supplementary Figure 1. Rates of YLLs and YLDs attributable to kidney dysfunction in categories of age-standardized, under 20, 20 to 54 years, and 55 plus in 21 countries in years 1990 and 2019.................................Page 26

Supplementary Figure 2. Attributed age-standardized rate of YLLs, YLDs, Deaths, and DALYs of kidney dysfunction of each country of the region in both sexes by SDI quintiles from 1990 to 2019..Page 27

Supplementary information. Authors’ contributions...Page 28

Supplementary Table 1. The SEV of North Africa and Middle East and 21 countries of the region in males, females, and both sexes in years 1990 and 2019 and percentage change of this two years

Location	Year						% Change (1990 to 2019)		
	1990			2019					
	Both	Female	Male	Both	Female	Male	Both	Female	Male
North Africa and Middle East	20.3 (14.6 to 27.4)	22.4 (16.3 to 29.9)	18.3 (12.9 to 25.1)	26.9 (20.4 to 34.6)	28.6 (21.9 to 36.3)	25.4 (19.1 to 32.7)	32.3 (23.1 to 44.0)	27.3 (19.8 to 36.9)	38.8 (27.1 to 53.7)
Afghanistan	19.8 (13.9 to 27.0)	22.1 (15.8 to 29.6)	17.2 (11.9 to 24.2)	25.0 (18.6 to 32.6)	27.3 (20.3 to 35.2)	22.8 (16.9 to 30.0)	26.7 (18.9 to 37.4)	23.2 (16.2 to 32.8)	32.5 (21.9 to 47.3)
Algeria	20.6 (14.8 to 27.7)	23.2 (16.9 to 30.6)	18.0 (12.7 to 24.9)	27.2 (20.8 to 34.9)	29.6 (22.6 to 37.4)	25.0 (18.8 to 32.4)	32.5 (23.3 to 45.5)	27.7 (19.8 to 38.5)	38.8 (26.7 to 55.9)
Bahrain	21.5 (15.7 to 28.7)	24.5 (18.0 to 32.0)	19.4 (14.0 to 26.3)	28.8 (22.3 to 36.1)	30.8 (23.8 to 38.7)	27.7 (21.4 to 35.0)	33.6 (23.7 to 46.0)	26.0 (18.2 to 36.5)	42.7 (29.7 to 59.3)
Egypt	20.5 (14.7 to 27.6)	23.4 (17.2 to 30.9)	17.5 (12.3 to 24.5)	28.1 (21.6 to 35.8)	30.7 (23.7 to 38.7)	25.9 (19.7 to 33.3)	37.5 (26.9 to 51.2)	31.0 (22.3 to 42.2)	47.8 (32.9 to 68.0)
Iran (Islamic Republic of)	22.8 (16.7 to 30.0)	24.7 (18.2 to 32.3)	21.0 (15.2 to 28.2)	26.2 (19.9 to 33.7)	28.1 (21.5 to 35.7)	24.4 (18.4 to 31.7)	15.0 (10.7 to 20.9)	13.9 (8.8 to 20.6)	16.2 (11.7 to 22.0)
Iraq	20.4 (14.7 to 27.6)	20.4 (14.7 to 27.6)	20.4 (14.7 to 27.6)	28.2 (21.5 to 35.8)	28.2 (21.5 to 35.8)	28.2 (21.5 to 35.8)	37.9 (26.6 to 53.6)	37.9 (26.6 to 53.6)	37.9 (26.6 to 53.6)
Jordan	18.8 (13.3 to 25.7)	18.8 (13.3 to 25.7)	18.8 (13.3 to 25.7)	27.1 (20.7 to 34.8)	27.1 (20.7 to 34.8)	27.1 (20.7 to 34.8)	43.7 (29.9 to 62.0)	43.7 (29.9 to 62.0)	43.7 (29.9 to 62.0)
Kuwait	19.7 (14.3 to 26.8)	19.7 (14.3 to 26.8)	19.7 (14.3 to 26.8)	25.1 (18.9 to 32.5)	25.1 (18.9 to 32.5)	25.1 (18.9 to 32.5)	27.6 (17.9 to 40.5)	27.6 (17.9 to 40.5)	27.6 (17.9 to 40.5)
Lebanon	18.3 (12.8 to 25.2)	18.3 (12.8 to 25.2)	18.3 (12.8 to 25.2)	26.6 (20.3 to 34.0)	26.6 (20.3 to 34.0)	26.6 (20.3 to 34.0)	44.8 (30.9 to 63.8)	44.8 (30.9 to 63.8)	44.8 (30.9 to 63.8)
Libya	18.7 (13.3 to 25.6)	18.7 (13.3 to 25.6)	18.7 (13.3 to 25.6)	25.3 (19.1 to 32.5)	25.3 (19.1 to 32.5)	25.3 (19.1 to 32.5)	35.1 (24.5 to 49.1)	35.1 (24.5 to 49.1)	35.1 (24.5 to 49.1)
Morocco	16.4 (11.2 to 23.2)	16.4 (11.2 to 23.2)	16.4 (11.2 to 23.2)	25.2 (18.9 to 32.7)	25.2 (18.9 to 32.7)	25.2 (18.9 to 32.7)	53.8 (36.5 to 77.7)	53.8 (36.5 to 77.7)	53.8 (36.5 to 77.7)
Oman	17.2 (12.0 to 24.0)	17.2 (12.0 to 24.0)	17.2 (12.0 to 24.0)	25.7 (19.3 to 33.0)	25.7 (19.3 to 33.0)	25.7 (19.3 to 33.0)	49.0 (33.2 to 69.7)	49.0 (33.2 to 69.7)	49.0 (33.2 to 69.7)
Palestine	20.0 (14.3 to 27.1)	20.0 (14.3 to 27.1)	20.0 (14.3 to 27.1)	26.9 (20.7 to 34.2)	26.9 (20.7 to 34.2)	26.9 (20.7 to 34.2)	34.3 (23.5 to 48.3)	34.3 (23.5 to 48.3)	34.3 (23.5 to 48.3)
Qatar	22.2 (16.4 to 29.4)	26.6 (20.2 to 34.1)	20.0 (14.3 to 27.0)	28.8 (22.4 to 36.2)	32.3 (25.5 to 40.0)	27.8 (21.4 to 35.1)	29.7 (20.9 to 41.2)	21.6 (14.7 to 30.1)	38.9 (27.0 to 55.0)
Saudi Arabia	22.1 (16.2 to 29.3)	25.5 (19.1 to 33.3)	19.8 (14.2 to 26.8)	31.9 (25.1 to 39.5)	34.8 (27.4 to 42.9)	30.0 (23.5 to 37.5)	44.1 (32.4 to 59.8)	36.5 (25.8 to 50.4)	51.3 (36.8 to 72.0)
Sudan	18.3 (12.8 to 25.3)	20.3 (14.4 to 27.5)	16.4 (11.1 to 23.2)	24.5 (18.3 to 32.0)	26.2 (19.7 to 33.7)	22.8 (16.9 to 29.9)	33.7 (23.3 to 48.0)	29.0 (20.5 to 40.6)	39.5 (26.3 to 57.4)
Syrian Arab Republic	21.1 (15.2 to 28.3)	23.8 (17.5 to 31.6)	18.6 (13.2 to 25.6)	26.7 (20.1 to 34.4)	28.7 (21.9 to 36.8)	24.7 (18.6 to 32.0)	26.6 (18.9 to 37.3)	20.6 (13.7 to 29.7)	32.3 (22.3 to 47.3)

SEV: Summary exposure value

Tunisia	19.8 (14.1 to 26.7)	22.0 (15.9 to 29.4)	17.5 (12.3 to 24.3)	26.6 (20.3 to 34.1)	28.3 (21.6 to 36.2)	24.8 (18.8 to 31.9)	34.7 (24.9 to 48.1)	28.7 (20.4 to 41.1)	41.4 (28.1 to 58.9)
Turkey	19.1 (13.4 to 26)	20.4 (14.5 to 27.8)	17.7 (12.3 to 24.5)	25.8 (19.7 to 33.2)	26.9 (20.4 to 34.5)	24.9 (18.7 to 32.2)	35.6 (24.7 to 51.1)	31.7 (21.5 to 46.6)	40.5 (27.5 to 59.3)
United Arab Emirates	23.5 (17.3 to 30.7)	26.9 (20.4 to 34.5)	21.7 (15.8 to 29.1)	29.7 (23.1 to 36.9)	32.7 (25.7 to 40.4)	28.6 (22.2 to 36.0)	26.4 (18.6 to 36.5)	21.3 (15.2 to 29.9)	32.2 (22.4 to 46.0)
Yemen	18.0 (12.6 to 25)	19.6 (13.8 to 26.7)	16.1 (11.0 to 22.9)	23.0 (16.9 to 30.4)	24.7 (18.0 to 32.7)	21.3 (15.5 to 28.5)	28.3 (19.2 to 40.9)	25.8 (16.3 to 40.8)	32.4 (21.4 to 46.5)

Data in parentheses are 95% Uncertainty Intervals (95% UIs)

Supplementary Table 2. The 21 countries of the region trend of epidemiologic indices attributed to kidney dysfunction, attributed number for all ages and age-standardized rates, for males, females, and both sexes, in 1990 and 2019 and percent of changes in the 1990-2019 period.

Country	Measure	Age, Metric	Year						% Change (1990 to 2019)		
			1990			2019					
			Both	Female	Male	Both	Female	Male	Both	Female	Male
Afghanistan	Deaths	Attributed all ages number	7503 (5836 to 9481)	3768 (2926 to 4849)	3735 (2863 to 4797)	12409 (9707 to 15641)	6870 (5313 to 9324)	5539 (4231 to 6924)	65.4 (28.1 to 105.7)	82.3 (37.5 to 136.1)	48.3 (14.5 to 81.8)
		Attributed age-standardized rate (per 100,000)	116.8 (90.6 to 150)	120.9 (93.3 to 157.4)	112.4 (85.8 to 146.2)	118.9 (92.4 to 150.3)	124.9 (95.5 to 166)	112.6 (85 to 142.5)	1.7 (-19.4 to 23.5)	3.2 (-20.3 to 29.9)	0.2 (-21.6 to 20.7)
	DALYs	Attributed all ages number	213838 (171624 to 265796)	114967 (90313 to 148482)	98871 (77779 to 123110)	390933 (310475 to 492218)	221945 (176071 to 296450)	168988 (132641 to 209778)	82.8 (44.6 to 127.3)	93.1 (47 to 151.2)	70.9 (36.6 to 112.3)
		Attributed age-standardized rate (per 100,000)	2744.2 (2165.1 to 3404.7)	2966.1 (2328.6 to 3779.6)	2498.4 (1942.2 to 3157.8)	2606.4 (2066.2 to 3288.1)	2825.5 (2220.8 to 3741.9)	2385.2 (1825.3 to 2958.1)	-5 (-26.1 to 17.7)	-4.7 (-27.7 to 21.6)	-4.5 (-24.9 to 16.7)
	YLLs	Attributed all ages number	203372 (159803 to 253528)	108389 (84089 to 141684)	94983 (74085 to 118704)	353090 (276206 to 454403)	198331 (153128 to 274341)	154759 (119202 to 193958)	73.6 (33.8 to 118.4)	83 (35.8 to 141.9)	62.9 (27.9 to 104.5)
		Attributed age-standardized rate (per 100,000)	2610 (2025 to 3270.3)	2799.5 (2170.6 to 3608.2)	2398.7 (1846.2 to 3045.1)	2410.8 (1867.7 to 3044.2)	2597.3 (1993.1 to 3528.3)	2221.6 (1677.4 to 2774.6)	-7.6 (-29.4 to 15.8)	-7.2 (-30.9 to 20.5)	-7.4 (-28.6 to 14.3)
	YLDs	Attributed all ages number	10466 (7644 to 13670)	6578 (4734 to 8590)	3888 (2785 to 5161)	37843 (27402 to 49655)	23614 (17183 to 30577)	14229 (9928 to 19320)	261.6 (227.6 to 297.6)	259 (221.4 to 299.2)	266 (221.4 to 317.9)
		Attributed age-standardized rate (per 100,000)	134.1 (98.5 to 175.4)	166.6 (121.4 to 216.6)	99.7 (71 to 132.8)	195.6 (144.1 to 255.3)	228.2 (168.5 to 295.4)	163.6 (116.8 to 221.5)	45.8 (37.3 to 55.4)	37 (27 to 47.7)	64 (50.4 to 79.3)
Algeria	Deaths	Attributed all ages number	8868 (7036 to 11146)	4599 (3610 to 6055)	4268 (3346 to 5331)	22541 (17735 to 27900)	11850 (9373 to 14646)	10691 (8177 to 13467)	154.2 (98.3 to 224.7)	157.6 (105 to 228)	150.5 (93.9 to 224.4)
		Attributed age-standardized rate (per 100,000)	105.7 (83.5 to 130.7)	118.4 (91.7 to 153.1)	97.5 (77.2 to 120.5)	90.4 (71.1 to 112.6)	105.8 (82.4 to 132.4)	80.7 (61.3 to 102.4)	-14.4 (-30.4 to 5.6)	-10.6 (-25.9 to 8.1)	-17.2 (-34.1 to 4.4)
	DALYs	Attributed all ages number	233935 (189441 to 292839)	125373 (99486 to 167508)	108562 (85815 to 134081)	498102 (406048 to 603068)	263114 (212826 to 322444)	234988 (186913 to 294661)	112.9 (71 to 166)	109.9 (63.4 to 163.8)	116.5 (70.5 to 176.8)
		Attributed age-standardized rate (per 100,000)	1917 (1542.5 to 2362.9)	2092.2 (1665.5 to 2709.4)	1781.2 (1420.9 to 2214.5)	1593.8 (1287.2 to 1944.3)	1769.7 (1433.8 to 2146.3)	1461.3 (1150.8 to 1818.6)	-16.9 (-32.6 to 2.4)	-15.4 (-31.2 to 3.8)	-18 (-34.6 to 3.9)

YLLs: Years of life lost, YLDs: Years lived with disability, DALYs: Disability-adjusted life years

The symbol −, used to denote that a number is negative and means decrease in % change

Country	Measure	Age, Metric	Year						% Change (1990 to 2019)		
			1990			2019					
			Both	Female	Male	Both	Female	Male	Both	Female	Male
	YLLs	Attributed all ages number	212159 (168734 to 270546)	112016 (87432 to 154487)	100143 (78318 to 125353)	424075 (336107 to 525245)	221987 (175485 to 276684)	202089 (156132 to 254452)	99.9 (55.6 to 155.8)	98.2 (50.1 to 155.1)	101.8 (54.5 to 165.3)
		Attributed age-standardized rate (per 100,000)	1776.3 (1412.3 to 2233.2)	1921.5 (1504.1 to 2549.4)	1669.7 (1314.5 to 2089.8)	1386.9 (1097.4 to 1722.9)	1541.1 (1225.5 to 1913.4)	1275.6 (976.1 to 1610.7)	-21.9 (-38.4 to -1.4)	-19.8 (-35.8 to 0.4)	-23.6 (-40.4 to -1.2)
	YLDs	Attributed all ages number	21776 (15890 to 28765)	13357 (9809 to 17652)	8419 (6023 to 11099)	74027 (53857 to 96897)	41128 (30281 to 53615)	32899 (23639 to 44073)	239.9 (210.5 to 270.6)	207.9 (177.6 to 239.4)	290.8 (247.5 to 336.2)
		Attributed age-standardized rate (per 100,000)	140.7 (103.6 to 184.3)	170.8 (126.2 to 226.4)	111.5 (80.3 to 148.1)	206.9 (151.5 to 274.5)	228.6 (169.8 to 299.2)	185.6 (133.9 to 249.9)	47 (36.9 to 57.9)	33.9 (22.4 to 45.6)	66.5 (51.6 to 83.5)
Bahrain	Deaths	Attributed all ages number	129 (105 to 155)	61 (50 to 73)	68 (54 to 84)	374 (294 to 461)	157 (126 to 193)	218 (170 to 273)	189.9 (128.7 to 265.9)	156.1 (101.2 to 225.1)	220.3 (147 to 312.6)
		Attributed age-standardized rate (per 100,000)	116 (93.2 to 140.7)	117.1 (94.4 to 141.7)	114.4 (90.6 to 141.3)	77.2 (60.9 to 94.6)	77 (61.5 to 93.9)	77.4 (60.7 to 96.8)	-33.4 (-46.2 to -17.6)	-34.2 (-47.4 to -18.3)	-32.3 (-46.3 to -14.9)
	DALYs	Attributed all ages number	3524 (2930 to 4167)	1601 (1343 to 1862)	1923 (1569 to 2333)	11036 (8984 to 13383)	4233 (3483 to 5061)	6802 (5406 to 8371)	213.1 (152.8 to 281.9)	164.3 (114.5 to 223.2)	253.7 (182.7 to 342.1)
		Attributed age-standardized rate (per 100,000)	2101.5 (1722.4 to 2506.2)	2147.6 (1781.7 to 2522.5)	2059.1 (1648.3 to 2533.8)	1378.6 (1111.8 to 1668.3)	1371.1 (1128.4 to 1653.4)	1388.2 (1116.2 to 1707.4)	-34.4 (-46.9 to -19.9)	-36.2 (-47.8 to -21.5)	-32.6 (-45.7 to -16.3)
	YLLs	Attributed all ages number	3123 (2574 to 3714)	1390 (1144 to 1643)	1733 (1381 to 2138)	8571 (6745 to 10553)	3248 (2600 to 4003)	5323 (4106 to 6752)	174.5 (114.2 to 247.5)	133.7 (81.6 to 198.8)	207.2 (136.8 to 299.9)
		Attributed age-standardized rate (per 100,000)	1948.1 (1585.6 to 2335.2)	1963.6 (1607.8 to 2333.4)	1929.7 (1537 to 2384.5)	1160.1 (919.7 to 1429.5)	1146.2 (922.8 to 1399.7)	1172.7 (920.4 to 1456.3)	-40.5 (-52.6 to -25.4)	-41.6 (-53.9 to -26.4)	-39.2 (-52.2 to -22.2)
	YLDs	Attributed all ages number	401 (286 to 525)	211 (153 to 277)	190 (133 to 254)	2465 (1748 to 3278)	985 (711 to 1280)	1479 (1028 to 1999)	513.9 (441.9 to 591.6)	366.2 (312.8 to 423)	678.1 (567 to 805.6)
		Attributed age-standardized rate (per 100,000)	153.4 (113.3 to 200.8)	184.1 (135.1 to 241)	129.4 (94.7 to 172.7)	218.5 (159.5 to 289.1)	225 (165.6 to 294.5)	215.5 (153.9 to 291.1)	42.4 (31 to 55)	22.2 (10.8 to 33.9)	66.5 (51 to 83.9)

Country	Measure	Age, Metric	Year						% Change (1990 to 2019)		
			1990			2019					
			Both	Female	Male	Both	Female	Male	Both	Female	Male
Egypt	Deaths	Attributed all ages number	23943 (19968 to 28343)	13537 (10498 to 16312)	10406 (8516 to 12441)	62497 (46551 to 80003)	30990 (21811 to 40366)	31506 (23097 to 40889)	161 (102.2 to 225.9)	128.9 (75.4 to 189.7)	202.8 (134.9 to 283.6)
		Attributed age-standardized rate (per 100,000)	102.8 (83.6 to 123)	117.3 (90.7 to 143.8)	88.2 (70.5 to 106.4)	128.7 (96.7 to 163.5)	162.5 (116.6 to 208.7)	110.9 (82.1 to 143.3)	25.1 (-1.3 to 53.5)	38.6 (8.8 to 71.4)	25.8 (-2 to 56.9)
	DALYs	Attributed all ages number	630780 (525670 to 731362)	353556 (273775 to 416356)	277224 (230090 to 327930)	1553642 (1168279 to 1958949)	768603 (554827 to 996990)	785039 (583826 to 1011242)	146.3 (98 to 205.2)	117.4 (74.7 to 170.5)	183.2 (125.9 to 254.3)
		Attributed age-standardized rate (per 100,000)	2078.7 (1728.5 to 2441.5)	2334.5 (1839.4 to 2768.7)	1819.3 (1502.4 to 2154.2)	2552.2 (1952.2 to 3196.4)	2956.2 (2158.6 to 3783.4)	2303.8 (1720.5 to 2942.9)	22.8 (-1.8 to 50.6)	26.6 (0.9 to 56.4)	26.6 (0.2 to 57.1)
	YLLs	Attributed all ages number	580611 (476384 to 678005)	322520 (243758 to 383591)	258092 (212452 to 307756)	1389393 (1009858 to 1803748)	681333 (471591 to 899406)	708060 (511502 to 928990)	139.3 (87 to 203.8)	111.3 (65.2 to 170.1)	174.3 (114 to 250.1)
		Attributed age-standardized rate (per 100,000)	1934.6 (1609.5 to 2282.4)	2157 (1669 to 2585.3)	1708.5 (1402.2 to 2029.4)	2319.1 (1723.5 to 2978.7)	2694.9 (1913.5 to 3502.5)	2095.3 (1533.1 to 2721.8)	19.9 (-6.7 to 49.7)	24.9 (-2.7 to 57.3)	22.6 (-5.1 to 55.2)
	YLDs	Attributed all ages number	50169 (36472 to 66176)	31036 (22700 to 40781)	19133 (13729 to 25356)	164249 (120241 to 213604)	87270 (63719 to 112922)	76979 (55530 to 102213)	227.4 (203.6 to 252.5)	181.2 (156.3 to 207.2)	302.3 (265.6 to 340.6)
		Attributed age-standardized rate (per 100,000)	144.1 (106.6 to 189.1)	177.6 (131.6 to 232.3)	110.8 (80.8 to 147.1)	233.2 (172 to 306.6)	261.3 (193.8 to 337.4)	208.6 (150 to 280)	61.8 (51.1 to 73.5)	47.2 (36.1 to 59.2)	88.3 (71.5 to 106.1)
Iran (Islamic Republic of)	Deaths	Attributed all ages number	15111 (12902 to 17507)	6868 (5843 to 7973)	8242 (6922 to 9598)	35987 (30559 to 41889)	17356 (14522 to 20245)	18631 (15793 to 21799)	138.2 (116.9 to 159.1)	152.7 (126.4 to 180.9)	126 (103.9 to 148.1)
		Attributed age-standardized rate (per 100,000)	78.4 (65 to 93.1)	75.1 (61.5 to 89.7)	80.8 (66.8 to 96.5)	58.2 (48.8 to 68.1)	58.2 (48 to 68.7)	58.4 (49 to 68.8)	-25.8 (-31.7 to -20.5)	-22.4 (-30.6 to -12.9)	-27.8 (-33.7 to -22)
	DALYs	Attributed all ages number	445178 (387847 to 502678)	200663 (176741 to 226572)	244514 (208327 to 281379)	790836 (692731 to 897947)	368995 (321289 to 415886)	421841 (367827 to 483023)	77.6 (64.2 to 91.3)	83.9 (69.6 to 100.3)	72.5 (57 to 90)
		Attributed age-standardized rate (per 100,000)	1596.7 (1377.8 to 1837.6)	1502.4 (1297 to 1733.1)	1673.4 (1419.8 to 1941.1)	1127.2 (981.1 to 1282.7)	1077.5 (933.4 to 1226.1)	1179.4 (1023.7 to 1351.6)	-29.4 (-33.9 to -24.7)	-28.3 (-33.9 to -22)	-29.5 (-35 to -24)

Country	Measure	Age, Metric	Year						% Change (1990 to 2019)		
			1990			2019					
			Both	Female	Male	Both	Female	Male	Both	Female	Male
	YLLs	Attributed all ages number	394008 (341358 to 447212)	172332 (150351 to 195320)	221675 (186173 to 255711)	662716 (579714 to 752950)	301612 (262948 to 341511)	361104 (314310 to 414065)	68.2 (55 to 82.8)	75 (58.6 to 93.2)	62.9 (46.9 to 81.1)
		Attributed age-standardized rate (per 100,000)	1445.4 (1233.3 to 1674.7)	1332.8 (1140.1 to 1542.8)	1540.5 (1297 to 1794.3)	959 (833.1 to 1097.4)	900.9 (773.5 to 1031.8)	1019.4 (881.4 to 1171)	-33.6 (-38.2 to -28.9)	-32.4 (-38.3 to -25.5)	-33.8 (-39.5 to -28.3)
	YLDs	Attributed all ages number	51170 (37040 to 66931)	28331 (20733 to 37146)	22839 (16492 to 29802)	128120 (93566 to 166807)	67383 (49342 to 87263)	60737 (43975 to 80304)	150.4 (131.6 to 170.3)	137.8 (119.6 to 157.4)	165.9 (143.9 to 190.7)
		Attributed age-standardized rate (per 100,000)	151.3 (112.5 to 197.2)	169.6 (125.6 to 219.8)	132.9 (97.7 to 173.7)	168.1 (123.8 to 219.8)	176.6 (130.4 to 231)	160 (116.9 to 210.6)	11.1 (5.6 to 16.9)	4.1 (-2.3 to 10.5)	20.3 (13.9 to 26.2)
Iraq	Deaths	Attributed all ages number	7531 (6178 to 9228)	3832 (3088 to 4881)	3699 (2918 to 4587)	20675 (16187 to 25018)	9299 (7323 to 11390)	11376 (8740 to 13976)	174.5 (117.5 to 233.8)	142.6 (90.6 to 208.9)	207.6 (136 to 286.8)
		Attributed age-standardized rate (per 100,000)	106.3 (86.7 to 131.2)	103.6 (82.4 to 132.4)	109.1 (85.6 to 137.2)	114.1 (89.6 to 136.3)	98.1 (77.9 to 119.1)	132.5 (103.8 to 161.2)	7.4 (-13.1 to 27.4)	-5.3 (-24.6 to 17.6)	21.5 (-4.6 to 48.4)
	DALYs	Attributed all ages number	200316 (169122 to 241281)	102220 (83533 to 128419)	98097 (78495 to 119788)	526446 (419950 to 640536)	238102 (191447 to 291416)	288344 (222869 to 353083)	162.8 (108.3 to 221.5)	132.9 (83.9 to 196.9)	193.9 (128.6 to 270.5)
		Attributed age-standardized rate (per 100,000)	2300.7 (1910.3 to 2783.4)	2276.9 (1860.4 to 2841.6)	2328 (1868.8 to 2844.4)	2306.7 (1839.2 to 2759.3)	2019.2 (1628.6 to 2444)	2620.2 (2041.7 to 3167.9)	0.3 (-19.6 to 20.6)	-11.3 (-30 to 12.1)	12.6 (-12.2 to 39.7)
	YLLs	Attributed all ages number	181985 (150500 to 221536)	91240 (73297 to 117332)	90745 (71205 to 111949)	454921 (350914 to 565967)	198795 (152835 to 250628)	256126 (194492 to 321929)	150 (92.9 to 214)	117.9 (66.1 to 186.7)	182.2 (112 to 264.1)
		Attributed age-standardized rate (per 100,000)	2118.7 (1737.7 to 2583.5)	2063.3 (1662.4 to 2622.1)	2178 (1723.4 to 2683.7)	2051.2 (1593.7 to 2494.1)	1747.1 (1359 to 2162.9)	2380.3 (1825.1 to 2927.7)	-3.2 (-23.9 to 18.8)	-15.3 (-34.8 to 10.1)	9.3 (-16.8 to 38.1)
	YLDs	Attributed all ages number	18331 (13481 to 23801)	10980 (8028 to 14250)	7352 (5359 to 9708)	71525 (51908 to 92782)	39307 (29112 to 50808)	32218 (22874 to 42855)	290.2 (261.2 to 321.3)	258 (223.8 to 291.2)	338.2 (295.2 to 385.8)
		Attributed age-standardized rate (per 100,000)	182 (135.8 to 236.1)	213.7 (159.1 to 280.5)	150 (108.9 to 197.6)	255.5 (189.3 to 330)	272.1 (200.7 to 347.7)	239.9 (172.6 to 322.5)	40.4 (31.7 to 49.8)	27.3 (17.5 to 37.5)	59.9 (47 to 75)

Country	Measure	Age, Metric	Year						% Change (1990 to 2019)		
			1990			2019					
			Both	Female	Male	Both	Female	Male	Both	Female	Male
Jordan	Deaths	Attributed all ages number	893 (759 to 1043)	507 (423 to 602)	385 (319 to 469)	3478 (2894 to 4179)	1643 (1316 to 2012)	1835 (1447 to 2330)	289.6 (219.6 to 374.9)	223.9 (149.3 to 313.5)	376.2 (261.8 to 526.7)
		Attributed age-standardized rate (per 100,000)	87.7 (73.9 to 103.4)	102.4 (84.6 to 122.8)	72.5 (59.9 to 88.1)	71.9 (59.2 to 86.4)	74.9 (60.1 to 91.7)	69.5 (54.2 to 87.7)	-18 (-32.2 to -1.6)	-26.9 (-42.9 to -8.2)	-4.1 (-26.3 to 24.8)
	DALYs	Attributed all ages number	24951 (21620 to 28713)	13701 (11727 to 16102)	11249 (9462 to 13368)	93878 (79584 to 110989)	43570 (36213 to 52117)	50308 (40432 to 62198)	276.3 (216.9 to 350.1)	218 (156.3 to 294.7)	347.2 (248.5 to 478.4)
		Attributed age-standardized rate (per 100,000)	1771.5 (1519.2 to 2067.8)	2022.3 (1713.3 to 2391.3)	1514.5 (1268.3 to 1820.9)	1461.5 (1236.3 to 1738.3)	1468 (1217.1 to 1748)	1454.9 (1164.5 to 1820.3)	-17.5 (-30.6 to -2.2)	-27.4 (-41.9 to -9.9)	-3.9 (-24.9 to 23.6)
	YLLs	Attributed all ages number	21803 (18672 to 25260)	11774 (9882 to 13892)	10029 (8387 to 12025)	75808 (63209 to 90640)	33924 (27036 to 42134)	41884 (32750 to 53098)	247.7 (181.4 to 329.8)	188.1 (118.1 to 278.4)	317.6 (215 to 457.6)
		Attributed age-standardized rate (per 100,000)	1600.3 (1358.2 to 1868.7)	1809 (1514.8 to 2143)	1384.2 (1146.3 to 1685.3)	1229 (1024.1 to 1476.6)	1211.4 (974 to 1487.3)	1243 (982.8 to 1574.7)	-23.2 (-37.4 to -6.1)	-33 (-48.4 to -13.8)	-10.2 (-31.7 to 19.3)
	YLDs	Attributed all ages number	3148 (2272 to 4125)	1928 (1393 to 2538)	1220 (868 to 1642)	18069 (12973 to 23690)	9646 (7014 to 12476)	8424 (5964 to 11297)	474.1 (429.9 to 524)	400.4 (353.5 to 450)	590.5 (516 to 675.7)
		Attributed age-standardized rate (per 100,000)	171.2 (127.1 to 223.2)	213.3 (158.9 to 278.8)	130.3 (94.3 to 173.4)	232.4 (169.7 to 304.4)	256.5 (187.7 to 331.5)	211.9 (152.4 to 283.8)	35.8 (26.4 to 46.4)	20.3 (10.4 to 30.9)	62.6 (47.9 to 79.6)
Kuwait	Deaths	Attributed all ages number	288 (248 to 328)	132 (112 to 151)	156 (134 to 179)	838 (676 to 1036)	258 (202 to 321)	580 (447 to 740)	191.3 (148.1 to 244.8)	96.2 (62.4 to 137.4)	271.6 (199 to 359.1)
		Attributed age-standardized rate (per 100,000)	61.1 (51.6 to 70.8)	68.3 (57.1 to 79.4)	54.7 (46 to 63.8)	42.1 (33.9 to 52.2)	33.4 (25.7 to 41.6)	47.9 (37.1 to 61.3)	-31.1 (-41 to -19)	-51.1 (-59.2 to -41)	-12.6 (-28.4 to 7.1)
	DALYs	Attributed all ages number	8917 (7851 to 9985)	3846 (3384 to 4318)	5071 (4387 to 5796)	24220 (19988 to 29335)	8366 (6839 to 10082)	15854 (12593 to 19853)	171.6 (136.5 to 212.7)	117.5 (87 to 154.1)	212.6 (159.5 to 274.1)
		Attributed age-standardized rate (per 100,000)	1276.5 (1107.7 to 1452.8)	1427.5 (1230.4 to 1623.2)	1165 (1000.9 to 1340.7)	905 (748 to 1103.7)	741.4 (611 to 884.8)	1013.9 (806.1 to 1264.3)	-29.1 (-38.5 to -17.8)	-48.1 (-55.9 to -39.1)	-13 (-27.6 to 4.6)

Country	Measure	Age, Metric	Year						% Change (1990 to 2019)		
			1990			2019					
			Both	Female	Male	Both	Female	Male	Both	Female	Male
	YLLs	Attributed all ages number	7533 (6610 to 8430)	3125 (2735 to 3519)	4408 (3794 to 5025)	18001 (14523 to 22244)	5312 (4239 to 6634)	12689 (9717 to 16221)	138.9 (101.3 to 185.7)	70 (39.4 to 109)	187.8 (128.8 to 258.7)
		Attributed age-standardized rate (per 100,000)	1135.2 (978 to 1297.2)	1253.9 (1075.2 to 1437.8)	1045 (894.6 to 1202.3)	717.3 (579.2 to 888.4)	545.5 (435.7 to 674.4)	834.7 (646 to 1070.9)	-36.8 (-46.3 to -24.7)	-56.5 (-64.2 to -46.9)	-20.1 (-35.7 to -1.1)
	YLDs	Attributed all ages number	1384 (978 to 1811)	721 (519 to 943)	663 (462 to 893)	6219 (4399 to 8292)	3054 (2171 to 4087)	3165 (2191 to 4310)	349.4 (302.8 to 398.1)	323.5 (271.5 to 386.1)	377.5 (313.6 to 443.6)
		Attributed age-standardized rate (per 100,000)	141.3 (103.7 to 185.8)	173.6 (128.2 to 224)	120 (87 to 161)	187.7 (136.4 to 248.9)	195.8 (141 to 253.7)	179.2 (128.1 to 242.8)	32.8 (21.1 to 45.3)	12.8 (2.5 to 24.4)	49.4 (33 to 66.7)
Lebanon	Deaths	Attributed all ages number	1570 (1274 to 1924)	772 (624 to 943)	799 (633 to 1001)	3995 (3037 to 4933)	1862 (1398 to 2290)	2133 (1593 to 2638)	154.4 (96.9 to 197.1)	141.3 (79.5 to 187.4)	167.1 (106.4 to 226.3)
		Attributed age-standardized rate (per 100,000)	84.8 (67.7 to 103.9)	81.4 (64.4 to 100.5)	88.2 (68.9 to 110.6)	78.4 (59.3 to 96.8)	66 (49.5 to 81.1)	93.7 (69.8 to 116)	-7.5 (-27.7 to 7.1)	-18.8 (-39.3 to -4.1)	6.3 (-17.3 to 28.4)
	DALYs	Attributed all ages number	36899 (30668 to 44548)	17822 (14714 to 21329)	19078 (15140 to 23621)	79074 (62032 to 95136)	36619 (28914 to 43944)	42455 (33022 to 52064)	114.3 (71.7 to 150.9)	105.5 (60.5 to 144.9)	122.5 (75.9 to 173.3)
		Attributed age-standardized rate (per 100,000)	1666.4 (1378.6 to 2017.1)	1591.7 (1309.6 to 1911.5)	1738.6 (1381.3 to 2153.2)	1516.5 (1193.6 to 1825.1)	1285.7 (1014.2 to 1546.1)	1798.9 (1396.5 to 2205)	-9 (-26.5 to 5.8)	-19.2 (-36.8 to -4.5)	3.5 (-17.5 to 25.6)
	YLLs	Attributed all ages number	33582 (27762 to 41076)	15792 (12819 to 19154)	17790 (14027 to 22196)	68420 (52111 to 83796)	30570 (22953 to 37490)	37850 (28664 to 47290)	103.7 (57.3 to 143.5)	93.6 (44.2 to 135.5)	112.8 (65 to 164.2)
		Attributed age-standardized rate (per 100,000)	1530 (1253.2 to 1873.6)	1427.7 (1156.9 to 1739.6)	1631.3 (1298 to 2035.6)	1314.2 (999.4 to 1607.7)	1071.8 (806.2 to 1314.6)	1608.6 (1219.1 to 2007.8)	-14.1 (-33.3 to 1.8)	-24.9 (-43.9 to -9.2)	-1.4 (-23.5 to 21.2)
	YLDs	Attributed all ages number	3317 (2422 to 4312)	2030 (1482 to 2656)	1288 (915 to 1720)	10654 (7785 to 13899)	6049 (4453 to 7794)	4605 (3295 to 6149)	221.2 (196.9 to 250.5)	198 (171.4 to 231.7)	257.6 (221.8 to 300.3)
		Attributed age-standardized rate (per 100,000)	136.4 (100.4 to 179)	163.9 (120.3 to 214.8)	107.2 (76.9 to 143.4)	202.3 (148.3 to 263)	213.9 (157.5 to 276.4)	190.3 (136.7 to 253.2)	48.3 (38.1 to 59.6)	30.5 (18.9 to 43.3)	77.4 (61 to 94.6)

Country	Measure	Age, Metric	Year						% Change (1990 to 2019)		
			1990			2019					
			Both	Female	Male	Both	Female	Male	Both	Female	Male
Libya	Deaths	Attributed all ages number	1044 (845 to 1267)	538 (428 to 665)	506 (395 to 626)	3174 (2485 to 3973)	1635 (1239 to 2073)	1540 (1161 to 2015)	204 (138.7 to 280.2)	203.6 (136 to 285.1)	204.4 (132.2 to 293.2)
		Attributed age-standardized rate (per 100,000)	62.5 (49.9 to 76.5)	66.3 (52.3 to 82.9)	59 (45.6 to 73.2)	72.3 (56.4 to 90.1)	74.7 (56.8 to 94.6)	69.9 (53.2 to 91.8)	15.7 (-8.8 to 44.5)	12.7 (-12.3 to 41.7)	18.4 (-9 to 51.3)
	DALYs	Attributed all ages number	27630 (23064 to 33145)	14418 (11865 to 17181)	13212 (10555 to 16183)	78607 (62727 to 96246)	41122 (32122 to 50981)	37485 (28940 to 47814)	184.5 (127.1 to 253.1)	185.2 (127.6 to 258.4)	183.7 (121 to 266.4)
		Attributed age-standardized rate (per 100,000)	1338.6 (1105.3 to 1599.2)	1468.3 (1204.5 to 1776.3)	1226 (972 to 1500.5)	1542.6 (1231.1 to 1881.8)	1634.6 (1282.3 to 2020.6)	1454.1 (1122.4 to 1849.3)	15.2 (-7.9 to 42.8)	11.3 (-11.5 to 39.4)	18.6 (-8 to 52.1)
	YLLs	Attributed all ages number	24275 (19814 to 29427)	12462 (10022 to 15119)	11812 (9253 to 14727)	66967 (51260 to 83901)	34623 (25950 to 44158)	32345 (24088 to 42470)	175.9 (113.3 to 256.3)	177.8 (112.4 to 263)	173.8 (106.8 to 265.4)
		Attributed age-standardized rate (per 100,000)	1200 (979 to 1458.3)	1297.5 (1034.6 to 1591.8)	1115.2 (870.9 to 1382.7)	1334.1 (1032.1 to 1654.2)	1400.7 (1060.4 to 1772.2)	1269.5 (956.8 to 1655.7)	11.2 (-13.6 to 41.9)	8 (-17.2 to 39.4)	13.8 (-14 to 49.3)
	YLDs	Attributed all ages number	3356 (2437 to 4427)	1956 (1431 to 2579)	1400 (989 to 1873)	11640 (8479 to 15241)	6499 (4744 to 8465)	5141 (3720 to 6836)	246.8 (216.6 to 278.9)	232.3 (196.2 to 273.4)	267.2 (228.4 to 309.6)
		Attributed age-standardized rate (per 100,000)	138.6 (101.7 to 181.7)	170.8 (126.8 to 225)	110.8 (79.5 to 146.9)	208.5 (153.6 to 274)	233.9 (171.3 to 305.3)	184.6 (134.1 to 249)	50.4 (40.7 to 61.3)	37 (25.2 to 49.4)	66.6 (52.4 to 82.7)
Morocco	Deaths	Attributed all ages number	8451 (7077 to 10074)	4305 (3550 to 5261)	4146 (3346 to 5030)	26331 (20738 to 31118)	12949 (10322 to 15533)	13383 (10063 to 16034)	211.6 (151.5 to 263.4)	200.8 (143.4 to 263.4)	222.8 (150.5 to 290)
		Attributed age-standardized rate (per 100,000)	73.4 (60.1 to 88.7)	72.6 (58.7 to 90.1)	74.8 (59 to 92)	104.1 (82.1 to 123.8)	99.4 (78.8 to 120.1)	109.1 (82.9 to 131)	41.7 (16.1 to 63.9)	36.9 (12 to 62.3)	46 (15 to 76.1)
	DALYs	Attributed all ages number	221004 (190717 to 256472)	114714 (97158 to 134509)	106291 (88195 to 126026)	600625 (477706 to 719484)	303020 (245568 to 365544)	297606 (226599 to 357267)	171.8 (121 to 220.3)	164.2 (114 to 223)	180 (120.2 to 238.3)
		Attributed age-standardized rate (per 100,000)	1536 (1305 to 1808)	1558.2 (1305.4 to 1857.3)	1516.4 (1244.5 to 1827.1)	2034.5 (1634.5 to 2399.2)	2000.3 (1622.6 to 2397.8)	2073.7 (1590.9 to 2466.5)	32.5 (9.9 to 54.2)	28.4 (4.9 to 53.8)	36.7 (8.3 to 64)

Country	Measure	Age, Metric	Year						% Change (1990 to 2019)		
			1990			2019					
			Both	Female	Male	Both	Female	Male	Both	Female	Male
	YLLs	Attributed all ages number	200823 (172439 to 235719)	102305 (85913 to 120941)	98518 (81071 to 118260)	530816 (407658 to 639609)	264257 (207445 to 321893)	266559 (197296 to 322475)	164.3 (109.1 to 216.4)	158.3 (103.5 to 222.6)	170.6 (107 to 231)
		Attributed age-standardized rate (per 100,000)	1414.4 (1195.6 to 1678)	1413.3 (1173.5 to 1716.3)	1419 (1154.6 to 1721.3)	1815.4 (1420.4 to 2163.1)	1762.7 (1397.6 to 2140.6)	1872.8 (1400.9 to 2246.6)	28.3 (3.3 to 51.1)	24.7 (-0.9 to 52.5)	32 (2.4 to 60.4)
	YLDs	Attributed all ages number	20181 (14578 to 26454)	12409 (9034 to 16265)	7772 (5522 to 10462)	69809 (50338 to 91514)	38763 (28263 to 50429)	31047 (22170 to 41582)	245.9 (217.3 to 274.7)	212.4 (181.6 to 245.1)	299.4 (260 to 341)
		Attributed age-standardized rate (per 100,000)	121.6 (89 to 159.6)	144.9 (106.2 to 189.6)	97.4 (69.5 to 131.2)	219.1 (159.1 to 285.3)	237.5 (174.6 to 310)	200.8 (144.5 to 269.1)	80.2 (68 to 92.3)	63.9 (50.2 to 78.3)	106.1 (87.8 to 127.3)
Oman	Deaths	Attributed all ages number	408 (313 to 526)	199 (152 to 258)	209 (156 to 275)	975 (810 to 1168)	441 (357 to 535)	534 (424 to 652)	138.8 (93.6 to 200.4)	121.4 (75.2 to 178.9)	155.3 (97.3 to 235.6)
		Attributed age-standardized rate (per 100,000)	88.4 (66.6 to 114.5)	86.6 (65.7 to 112.5)	91.4 (68 to 118.9)	104.4 (84.4 to 127.7)	98.2 (78 to 121.3)	112.7 (88.4 to 138.1)	18 (-2.9 to 44.6)	13.4 (-9.4 to 40.3)	23.4 (-2.5 to 54.8)
	DALYs	Attributed all ages number	10738 (8455 to 13608)	4947 (3906 to 6170)	5791 (4418 to 7508)	26188 (22166 to 30568)	11343 (9545 to 13470)	14846 (12095 to 17722)	143.9 (97.4 to 203.8)	129.3 (86 to 183.7)	156.4 (100.8 to 230.9)
		Attributed age-standardized rate (per 100,000)	1705.9 (1320.2 to 2182.6)	1697.2 (1322.8 to 2152)	1722.7 (1303.7 to 2230)	1818.8 (1520.9 to 2163)	1788.4 (1473.2 to 2132.2)	1879.7 (1507.3 to 2275)	6.6 (-12.3 to 31.9)	5.4 (-14.7 to 29.6)	9.1 (-13.4 to 38.7)
	YLLs	Attributed all ages number	9486 (7294 to 12235)	4298 (3304 to 5505)	5187 (3851 to 6856)	20530 (17248 to 24285)	8909 (7328 to 10718)	11621 (9325 to 14080)	116.4 (71.4 to 178.5)	107.3 (63.1 to 167.3)	124 (71.6 to 199.5)
		Attributed age-standardized rate (per 100,000)	1570.2 (1200.1 to 2021.4)	1535.5 (1177.3 to 1975.6)	1609.1 (1197 to 2103.7)	1588.5 (1314.3 to 1917.1)	1533.7 (1232.8 to 1861.8)	1665.2 (1318.4 to 2029.9)	1.2 (-17.8 to 27.6)	-0.1 (-21.6 to 25.9)	3.5 (-19.2 to 33.8)
	YLDs	Attributed all ages number	1252 (903 to 1640)	649 (473 to 850)	604 (429 to 810)	5658 (4060 to 7474)	2433 (1770 to 3197)	3225 (2259 to 4334)	351.7 (308.8 to 403.3)	275 (235 to 319.3)	434.2 (368.9 to 509.4)
		Attributed age-standardized rate (per 100,000)	135.7 (100 to 176.8)	161.7 (118.9 to 209.4)	113.6 (83.5 to 150.2)	230.3 (170.9 to 303.3)	254.7 (190.9 to 335)	214.5 (155.8 to 285.7)	69.7 (58.4 to 82.5)	57.6 (45.5 to 71.1)	88.8 (70.5 to 108.9)

Country	Measure	Age, Metric	Year						% Change (1990 to 2019)		
			1990			2019					
			Both	Female	Male	Both	Female	Male	Both	Female	Male
Palestine	Deaths	Attributed all ages number	820 (649 to 1024)	421 (331 to 522)	399 (315 to 508)	1653 (1380 to 1957)	822 (682 to 975)	831 (687 to 991)	101.5 (62.8 to 152.5)	95.3 (57.3 to 146.5)	108.1 (65 to 165.6)
		Attributed age-standardized rate (per 100,000)	104.7 (82.4 to 130.7)	97.1 (75.9 to 120.9)	115.1 (90.7 to 145.4)	91.7 (75.7 to 109.7)	83.5 (68.7 to 99.9)	105.3 (86.5 to 125.9)	-12.4 (-28.8 to 8.6)	-14 (-30.7 to 8.4)	-8.5 (-26.9 to 14.9)
	DALYs	Attributed all ages number	20989 (17059 to 25937)	10937 (8915 to 13294)	10052 (8008 to 12588)	42541 (36524 to 49117)	20283 (17448 to 23438)	22258 (18884 to 26046)	102.7 (64.7 to 150.1)	85.4 (52.5 to 128.3)	121.4 (76.5 to 181)
		Attributed age-standardized rate (per 100,000)	2161 (1727.7 to 2691.6)	2045.3 (1640.6 to 2518.3)	2317.5 (1830.4 to 2945.1)	1818.2 (1535.3 to 2123.8)	1664.5 (1413.8 to 1943.5)	2010.6 (1680.8 to 2389.8)	-15.9 (-31.7 to 4.6)	-18.6 (-33.5 to 0.8)	-13.2 (-30.8 to 9.7)
	YLLs	Attributed all ages number	19088 (15193 to 23871)	9790 (7849 to 12106)	9298 (7263 to 11843)	35921 (30129 to 42291)	16658 (14062 to 19530)	19263 (16094 to 22814)	88.2 (48.6 to 138.5)	70.1 (36.9 to 114.8)	107.2 (61 to 168.3)
		Attributed age-standardized rate (per 100,000)	1998.4 (1569.2 to 2516.1)	1859 (1454.5 to 2321.5)	2181.5 (1705.6 to 2789)	1597.9 (1328 to 1892)	1433.1 (1201.1 to 1693.3)	1801 (1494.1 to 2147.5)	-20 (-36.2 to 1.7)	-22.9 (-38.3 to -1.8)	-17.4 (-35.4 to 6.4)
	YLDs	Attributed all ages number	1901 (1382 to 2497)	1147 (839 to 1495)	754 (533 to 1007)	6621 (4836 to 8690)	3625 (2669 to 4757)	2996 (2136 to 4015)	248.3 (222.1 to 278.2)	216.1 (184.7 to 249.1)	297.2 (252.2 to 344.9)
		Attributed age-standardized rate (per 100,000)	162.6 (119.6 to 211.8)	186.3 (137.2 to 243.8)	136 (99 to 179.1)	220.3 (161.7 to 289.1)	231.4 (172.2 to 300.1)	209.6 (153.1 to 280)	35.5 (26.6 to 44.9)	24.2 (13.3 to 35.5)	54.1 (40.6 to 70.9)
Qatar	Deaths	Attributed all ages number	70 (56 to 87)	31 (24 to 40)	38 (30 to 49)	307 (231 to 396)	106 (84 to 133)	201 (147 to 266)	340.2 (233.7 to 479.6)	240 (156.3 to 338)	421.4 (274.5 to 630.3)
		Attributed age-standardized rate (per 100,000)	124.6 (99.4 to 155.8)	132.2 (103.4 to 173.9)	119.7 (90.7 to 153.2)	113.6 (88.7 to 142.1)	164.3 (131.1 to 202.3)	98 (73.6 to 125.1)	-8.9 (-28.6 to 12.8)	24.3 (-5.6 to 53.9)	-18.1 (-37.9 to 6.7)
	DALYs	Attributed all ages number	2111 (1737 to 2548)	855 (694 to 1061)	1257 (1002 to 1560)	11343 (9148 to 13992)	3539 (2869 to 4265)	7805 (6157 to 9764)	437.3 (324.9 to 571.6)	314.1 (230.2 to 406.3)	521 (371.1 to 711.3)
		Attributed age-standardized rate (per 100,000)	2171.4 (1771 to 2675.2)	2401.9 (1920.4 to 3088)	2046 (1597.8 to 2589.6)	1750.5 (1384.1 to 2183.4)	2450.9 (2009.1 to 2962.5)	1521.3 (1186.8 to 1922.3)	-19.4 (-35.4 to -0.7)	2 (-19.6 to 25.9)	-25.6 (-43.4 to -4.2)

Country	Measure	Age, Metric	Year						% Change (1990 to 2019)		
			1990			2019					
			Both	Female	Male	Both	Female	Male	Both	Female	Male
	YLLs	Attributed all ages number	1785 (1448 to 2204)	719 (567 to 924)	1066 (823 to 1356)	7657 (5692 to 9832)	2453 (1905 to 3120)	5205 (3814 to 6832)	328.9 (218.2 to 470)	241.2 (156.7 to 342.1)	388.1 (250.2 to 584.9)
		Attributed age-standardized rate (per 100,000)	2008.5 (1619.6 to 2506.1)	2196.6 (1720 to 2866.9)	1907.6 (1468 to 2439.2)	1526.1 (1188.2 to 1922.7)	2206.9 (1788.2 to 2713.7)	1303 (988.3 to 1677.3)	-24 (-40.6 to -4.2)	0.5 (-23 to 26.5)	-31.7 (-49.1 to -9.1)
	YLDs	Attributed all ages number	326 (226 to 437)	136 (97 to 179)	190 (128 to 262)	3686 (2527 to 4981)	1086 (754 to 1455)	2600 (1767 to 3552)	1030 (910.5 to 1169.4)	700 (591.9 to 810.9)	1265.3 (1078.4 to 1490.4)
		Attributed age-standardized rate (per 100,000)	162.9 (120.3 to 219)	205.3 (151.7 to 271.9)	138.5 (100.8 to 187.2)	224.4 (163.3 to 298.1)	243.9 (179.3 to 309.1)	218.4 (156 to 295.5)	37.7 (26.2 to 50.1)	18.8 (7.8 to 30)	57.7 (43.1 to 73.9)
Saudi Arabia	Deaths	Attributed all ages number	4723 (3804 to 5702)	2082 (1681 to 2522)	2641 (2057 to 3248)	14298 (11391 to 17476)	5703 (4446 to 7246)	8595 (6961 to 10481)	202.7 (130.5 to 289.4)	173.9 (101.7 to 264.6)	225.5 (146.6 to 325.2)
		Attributed age-standardized rate (per 100,000)	96.9 (78.7 to 116.1)	96.4 (77.9 to 116.8)	98 (76.6 to 118.8)	112.8 (92.7 to 135.1)	108.5 (85.6 to 136.2)	115.8 (95.3 to 137.4)	16.4 (-7.8 to 45.8)	12.6 (-15.5 to 46.8)	18.1 (-7.6 to 50.1)
	DALYs	Attributed all ages number	130865 (105931 to 159381)	58030 (47093 to 69939)	72835 (56827 to 89963)	450378 (362729 to 552629)	187050 (148917 to 232651)	263328 (212134 to 323717)	244.2 (163.6 to 339.2)	222.3 (144.8 to 318.5)	261.5 (174.3 to 372.5)
		Attributed age-standardized rate (per 100,000)	2040.6 (1654.5 to 2456.4)	2104.9 (1713.2 to 2533)	2003.7 (1575.1 to 2462.5)	2369.7 (1935.6 to 2834.4)	2346.2 (1879.4 to 2903.7)	2384.2 (1966.4 to 2846.9)	16.1 (-9.3 to 45.7)	11.5 (-15.2 to 44.5)	19 (-6.9 to 51.5)
	YLLs	Attributed all ages number	117633 (92951 to 145363)	51201 (40229 to 62904)	66432 (51105 to 83587)	385818 (302141 to 483793)	156547 (120694 to 201537)	229271 (180595 to 286443)	228 (142.2 to 334.6)	205.8 (119.7 to 319.9)	245.1 (154.9 to 365.9)
		Attributed age-standardized rate (per 100,000)	1880.8 (1497.4 to 2288.7)	1909.9 (1526.9 to 2336.5)	1869.2 (1451 to 2312.4)	2104.5 (1701.8 to 2552)	2057.4 (1624.8 to 2586.1)	2135.1 (1738.7 to 2559.6)	11.9 (-14.2 to 44.1)	7.7 (-20.9 to 43.7)	14.2 (-12.9 to 48.7)
	YLDs	Attributed all ages number	13232 (9437 to 17283)	6829 (4927 to 8837)	6403 (4518 to 8536)	64560 (45265 to 85628)	30503 (21544 to 40333)	34057 (23872 to 45557)	387.9 (336.2 to 449.1)	346.7 (286.5 to 418.1)	431.9 (369.6 to 505.2)
		Attributed age-standardized rate (per 100,000)	159.8 (117.9 to 206.5)	195 (144.2 to 252.5)	134.5 (98 to 176)	265.1 (194.9 to 348.4)	288.9 (212.1 to 375.9)	249.1 (180.2 to 331.2)	65.9 (53.2 to 79.7)	48.1 (34.5 to 64.1)	85.2 (68.5 to 103.5)

Country	Measure	Age, Metric	Year						% Change (1990 to 2019)		
			1990			2019					
			Both	Female	Male	Both	Female	Male	Both	Female	Male
Sudan	Deaths	Attributed all ages number	6694 (5402 to 8134)	3208 (2607 to 3934)	3486 (2785 to 4299)	13743 (10771 to 17360)	6155 (4931 to 7612)	7589 (5776 to 10013)	105.3 (63.7 to 158)	91.9 (48.7 to 140.1)	117.7 (67.5 to 186.8)
		Attributed age-standardized rate (per 100,000)	80.4 (64.1 to 98.5)	79.3 (63 to 98.4)	81.5 (63.4 to 101.8)	88.5 (70 to 111.6)	86.2 (68.6 to 107)	90.6 (68.6 to 118.5)	10.1 (-10.5 to 36)	8.7 (-13.3 to 32.4)	11.1 (-13.3 to 42.8)
	DALYs	Attributed all ages number	189950 (154102 to 230487)	95375 (76528 to 120404)	94575 (74236 to 116131)	354428 (278503 to 444020)	165074 (131461 to 202847)	189355 (143364 to 247837)	86.6 (46.1 to 142.6)	73.1 (25.7 to 122.3)	100.2 (52.9 to 175.1)
		Attributed age-standardized rate (per 100,000)	1756 (1422.1 to 2122.3)	1774.8 (1461 to 2144.9)	1736.5 (1400.3 to 2133.4)	1801.4 (1437.3 to 2253.7)	1775.7 (1449.7 to 2142.4)	1821.2 (1390.1 to 2368.3)	2.6 (-17.7 to 27.9)	0.1 (-20.8 to 23.6)	4.9 (-19 to 37.5)
	YLLs	Attributed all ages number	176447 (142575 to 215550)	87306 (68847 to 111081)	89140 (69396 to 110289)	309498 (238645 to 395269)	140146 (108876 to 176626)	169352 (125696 to 223852)	75.4 (32.8 to 134.4)	60.5 (11.5 to 113)	90 (40.6 to 168)
		Attributed age-standardized rate (per 100,000)	1641.7 (1322.5 to 1998)	1637 (1327.1 to 2011.6)	1645 (1312.9 to 2029.9)	1615.5 (1267.2 to 2068.3)	1568.7 (1256.2 to 1955.7)	1655.4 (1245.6 to 2193)	-1.6 (-23 to 25.5)	-4.2 (-26.3 to 21)	0.6 (-24 to 35.1)
	YLDs	Attributed all ages number	13503 (9857 to 17756)	8069 (5896 to 10637)	5435 (3910 to 7167)	44930 (32249 to 59166)	24927 (18083 to 32654)	20003 (14428 to 27043)	232.7 (211.4 to 257.2)	208.9 (179.5 to 240.4)	268.1 (236.1 to 306.2)
		Attributed age-standardized rate (per 100,000)	114.3 (84.3 to 149.9)	137.8 (101.8 to 183.4)	91.5 (66.6 to 120.2)	185.9 (136.3 to 244.3)	207.1 (152.8 to 271.9)	165.7 (121 to 224.1)	62.7 (53 to 73.3)	50.3 (38.3 to 61.8)	81.2 (65.3 to 98.8)
Syrian Arab Republic	Deaths	Attributed all ages number	4700 (3782 to 5766)	2320 (1852 to 2847)	2380 (1888 to 2983)	9880 (7409 to 13016)	4648 (3521 to 6044)	5231 (3858 to 6960)	110.2 (58.1 to 178.6)	100.4 (52.8 to 167.1)	119.8 (59.2 to 202.6)
		Attributed age-standardized rate (per 100,000)	100.3 (79.6 to 124.4)	107.6 (84.6 to 134.1)	94 (73.3 to 119.6)	109.4 (82.2 to 141.7)	123.5 (92.6 to 156.5)	104.5 (77.3 to 136.7)	9 (-16 to 41.9)	14.8 (-10.1 to 48.4)	11.2 (-17.6 to 51.3)
	DALYs	Attributed all ages number	134378 (111717 to 161098)	65512 (54185 to 78772)	68866 (56022 to 84184)	230986 (176426 to 303005)	108228 (83521 to 138828)	122757 (91693 to 163325)	71.9 (30.7 to 130)	65.2 (26.7 to 120.1)	78.3 (32.7 to 143.8)
		Attributed age-standardized rate (per 100,000)	2143.1 (1755.2 to 2596.1)	2204.4 (1797.7 to 2679.2)	2088.8 (1669.7 to 2604.4)	2033.8 (1570.3 to 2622.2)	2087.6 (1637.4 to 2646.7)	2045.8 (1547.9 to 2694.8)	-5.1 (-27.7 to 24.5)	-5.3 (-25.9 to 23.9)	-2.1 (-27 to 31.9)

Country	Measure	Age, Metric	Year						% Change (1990 to 2019)		
			1990			2019					
			Both	Female	Male	Both	Female	Male	Both	Female	Male
	YLLs	Attributed all ages number	122775 (100390 to 149628)	58645 (47896 to 71233)	64130 (51183 to 79438)	206242 (152346 to 276339)	94721 (71088 to 125582)	111522 (80899 to 149891)	68 (23.8 to 132.4)	61.5 (19.3 to 123.3)	73.9 (25.7 to 145.3)
		Attributed age-standardized rate (per 100,000)	1986.1 (1606.7 to 2439.5)	2014 (1617.2 to 2477.9)	1962.6 (1546.6 to 2480.9)	1834.1 (1376.7 to 2424.8)	1871.2 (1416.9 to 2417.8)	1864.4 (1370 to 2497.8)	-7.6 (-31.1 to 24.4)	-7.1 (-29.4 to 25)	-5 (-31 to 31.1)
	YLDs	Attributed all ages number	11603 (8383 to 15421)	6867 (4966 to 9147)	4735 (3363 to 6314)	24743 (18057 to 32368)	13508 (9902 to 17668)	11236 (8055 to 15206)	113.3 (91.4 to 139.2)	96.7 (75.1 to 122.3)	137.3 (107.2 to 174.3)
		Attributed age-standardized rate (per 100,000)	157 (115 to 205)	190.3 (140.4 to 247.9)	126.2 (90.8 to 166.3)	199.6 (146.4 to 262.6)	216.4 (159.6 to 283.2)	181.4 (129.9 to 244.3)	27.1 (18.2 to 37.5)	13.7 (4.1 to 24.5)	43.7 (29.7 to 59.7)
Tunisia	Deaths	Attributed all ages number	2573 (2135 to 3074)	1203 (1002 to 1451)	1370 (1110 to 1671)	7956 (5842 to 10301)	3797 (2830 to 4844)	4158 (3008 to 5506)	209.1 (134.4 to 304.1)	215.6 (138.5 to 300.6)	203.4 (122.3 to 311.5)
		Attributed age-standardized rate (per 100,000)	64.5 (52.7 to 77.8)	62.3 (51 to 76.3)	66.9 (53.4 to 81.6)	72.3 (53.2 to 93.2)	64.9 (48.1 to 82.6)	80.9 (58.6 to 106.6)	12.1 (-14.1 to 44)	4.2 (-20.7 to 31.8)	20.8 (-9.3 to 60.5)
	DALYs	Attributed all ages number	64027 (55209 to 74593)	30983 (26763 to 36185)	33044 (27559 to 39224)	163186 (125397 to 208082)	76383 (59745 to 96074)	86803 (65060 to 113859)	154.9 (97.8 to 227.8)	146.5 (92.7 to 209.4)	162.7 (98.8 to 250.6)
		Attributed age-standardized rate (per 100,000)	1252.6 (1065.8 to 1468.3)	1236.1 (1043.9 to 1463.2)	1270.6 (1045.2 to 1524.9)	1364.1 (1044.9 to 1730.8)	1233.8 (966.3 to 1550.5)	1506.1 (1135.4 to 1974.4)	8.9 (-14.8 to 39.2)	-0.2 (-21.6 to 25.3)	18.5 (-9.3 to 57.5)
	YLLs	Attributed all ages number	56930 (48491 to 66977)	27021 (22990 to 31995)	29909 (24516 to 36102)	140454 (102772 to 185570)	64519 (47973 to 83614)	75935 (54447 to 102226)	146.7 (83.8 to 228.7)	138.8 (78.3 to 211.2)	153.9 (84 to 250)
		Attributed age-standardized rate (per 100,000)	1131.8 (948.9 to 1345.4)	1100.9 (924.8 to 1321.3)	1164.1 (944.8 to 1413.9)	1178.1 (862.9 to 1541.9)	1045 (776 to 1349.8)	1322.7 (956.1 to 1762.3)	4.1 (-22 to 37.3)	-5.1 (-29.2 to 23)	13.6 (-16.5 to 55.5)
	YLDs	Attributed all ages number	7097 (5172 to 9366)	3962 (2851 to 5252)	3135 (2244 to 4125)	22732 (16675 to 29642)	11864 (8773 to 15368)	10868 (7871 to 14524)	220.3 (192.9 to 249.5)	199.4 (169.3 to 232.9)	246.7 (213.6 to 283.1)
		Attributed age-standardized rate (per 100,000)	120.8 (88.6 to 157.1)	135.2 (99.4 to 177.9)	106.5 (77.8 to 139.3)	186 (136.6 to 243.5)	188.8 (140.3 to 245)	183.4 (132.9 to 245.2)	54 (43.6 to 65.8)	39.6 (27.8 to 53)	72.2 (57.4 to 88.5)

Country	Measure	Age, Metric	Year						% Change (1990 to 2019)		
			1990			2019					
			Both	Female	Male	Both	Female	Male	Both	Female	Male
Turkey	Deaths	Attributed all ages number	21481 (17796 to 26473)	9956 (8161 to 12796)	11526 (9146 to 14697)	43971 (34537 to 55268)	22423 (17526 to 28202)	21548 (16734 to 27013)	104.7 (53.4 to 159.1)	125.2 (65.6 to 188.7)	87 (35.9 to 147.2)
		Attributed age-standardized rate (per 100,000)	67.6 (55.3 to 84.3)	58.7 (47.6 to 77.2)	77.8 (60.9 to 101.8)	54.3 (42.6 to 68)	50.1 (39.2 to 62.9)	59 (45.9 to 73.3)	-19.7 (-40.3 to 2)	-14.7 (-38 to 8.8)	-24.2 (-46 to 0.8)
	DALYs	Attributed all ages number	569570 (491028 to 667204)	257550 (217653 to 313092)	312020 (256553 to 373828)	912927 (746267 to 1115254)	437706 (360681 to 531911)	475221 (384359 to 582714)	60.3 (27.2 to 98.3)	69.9 (32.5 to 111)	52.3 (18.3 to 96.3)
		Attributed age-standardized rate (per 100,000)	1472.8 (1247.1 to 1762.3)	1276.2 (1066.6 to 1570.4)	1686.8 (1368.2 to 2077.9)	1075.1 (880.1 to 1313.9)	968.1 (800 to 1174.9)	1191.6 (965.3 to 1454.8)	-27 (-42.8 to -9.3)	-24.1 (-40.9 to -6.4)	-29.4 (-46 to -8.5)
	YLLs	Attributed all ages number	517326 (441903 to 613247)	227652 (189676 to 282198)	289674 (236552 to 349551)	757662 (598631 to 953925)	354745 (281792 to 446657)	402917 (314887 to 508438)	46.5 (12 to 87)	55.8 (16.6 to 100.1)	39.1 (4.5 to 83.6)
		Attributed age-standardized rate (per 100,000)	1348.1 (1134.3 to 1631)	1138.9 (947.1 to 1422.4)	1575 (1269.6 to 1964.2)	895.3 (710.2 to 1124.5)	784.2 (624.9 to 984.2)	1015.1 (795.3 to 1270.8)	-33.6 (-49.8 to -15.2)	-31.1 (-48.8 to -11.6)	-35.5 (-52.5 to -14.3)
	YLDs	Attributed all ages number	52244 (38785 to 69288)	29898 (22049 to 39774)	22346 (16201 to 29268)	155265 (113037 to 203431)	82961 (60938 to 108032)	72304 (50805 to 96230)	197.2 (169.8 to 227.4)	177.5 (149.4 to 212.8)	223.6 (186 to 266.2)
		Attributed age-standardized rate (per 100,000)	124.8 (92 to 163.6)	137.3 (101.9 to 182.2)	111.8 (81.3 to 147)	179.8 (132.3 to 235.4)	184 (135.1 to 240.2)	176.5 (125.9 to 235)	44.1 (33 to 56.8)	34 (21.7 to 49.4)	57.9 (41.5 to 75.8)
United Arab Emirates	Deaths	Attributed all ages number	358 (295 to 431)	124 (99 to 155)	234 (181 to 295)	2415 (1723 to 3412)	542 (382 to 710)	1872 (1260 to 2783)	574 (374.2 to 844.9)	335.9 (194.4 to 505.4)	700.5 (448.3 to 1094.1)
		Attributed age-standardized rate (per 100,000)	127.7 (105.9 to 150.6)	135.1 (108.9 to 166.1)	121.8 (97 to 148.4)	98.5 (74.8 to 128.4)	98.3 (72.2 to 126.6)	98.5 (73 to 135.6)	-22.9 (-39.3 to -1.7)	-27.2 (-46 to -6.7)	-19.1 (-39 to 8)
	DALYs	Attributed all ages number	13282 (10983 to 16115)	4275 (3432 to 5333)	9008 (7088 to 11408)	99060 (72675 to 137741)	21974 (16129 to 28543)	77086 (53982 to 113701)	645.8 (442.1 to 913.8)	414 (255.2 to 582.9)	755.8 (501.9 to 1125.7)
		Attributed age-standardized rate (per 100,000)	2537.4 (2142.1 to 2959.1)	2623.6 (2134.9 to 3200.1)	2466.5 (1985 to 3006.4)	2074.4 (1614.2 to 2673.5)	2028 (1547.7 to 2525.3)	2088.4 (1559 to 2852.4)	-18.2 (-36.2 to 4.4)	-22.7 (-43.1 to -1.3)	-15.3 (-36.3 to 12.9)

Country	Measure	Age, Metric	Year						% Change (1990 to 2019)		
			1990			2019					
			Both	Female	Male	Both	Female	Male	Both	Female	Male
	YLLs	Attributed all ages number	11721 (9514 to 14467)	3636 (2842 to 4652)	8084 (6231 to 10448)	84078 (58260 to 122969)	17643 (12039 to 23810)	66435 (43807 to 102467)	617.3 (389.3 to 922.4)	385.2 (206.2 to 586.4)	721.8 (446.8 to 1135)
		Attributed age-standardized rate (per 100,000)	2349.8 (1948.7 to 2756.6)	2394.8 (1917.6 to 2952)	2302.7 (1834.1 to 2824.7)	1818.4 (1374.4 to 2408.5)	1746.7 (1274.5 to 2248.3)	1842.5 (1336.4 to 2601.5)	-22.6 (-41.6 to 1.6)	-27.1 (-48.2 to -3.5)	-20 (-41.4 to 9.8)
	YLDs	Attributed all ages number	1562 (1100 to 2074)	638 (460 to 830)	923 (630 to 1272)	14982 (10294 to 20148)	4331 (3078 to 5832)	10651 (7186 to 14496)	859.4 (728.1 to 997.6)	578.5 (481.6 to 698.6)	1053.6 (869.6 to 1263.2)
		Attributed age-standardized rate (per 100,000)	187.5 (138.9 to 245.5)	228.9 (169 to 295.8)	163.8 (118.4 to 218.2)	256 (187.4 to 337.5)	281.3 (208.4 to 367.1)	246 (178.1 to 333.3)	36.5 (27.3 to 46.4)	22.9 (13.9 to 33.1)	50.2 (37.9 to 64.7)
Yemen	Deaths	Attributed all ages number	3072 (2379 to 3957)	1576 (1231 to 2020)	1495 (1117 to 1994)	8832 (6922 to 11353)	4227 (3281 to 5530)	4605 (3581 to 5880)	187.5 (128.6 to 264.8)	168.2 (111.4 to 243.3)	208 (141.4 to 304.4)
		Attributed age-standardized rate (per 100,000)	75.1 (58.2 to 97.1)	70.6 (54.6 to 92)	81.4 (60.9 to 106.5)	83.7 (66.1 to 106.5)	78.6 (61 to 102.6)	89.3 (69.6 to 113.2)	11.4 (-9.2 to 37.9)	11.3 (-10.2 to 40)	9.7 (-12 to 39.4)
	DALYs	Attributed all ages number	92359 (71874 to 118719)	46487 (35891 to 61615)	45873 (33807 to 60307)	239059 (189628 to 304157)	117413 (92230 to 151274)	121646 (94436 to 156518)	158.8 (104.3 to 234.9)	152.6 (94 to 231.4)	165.2 (107.3 to 255)
		Attributed age-standardized rate (per 100,000)	1629.6 (1269.9 to 2099)	1552.1 (1226.7 to 1998)	1721.7 (1298.8 to 2260.7)	1715.5 (1368.2 to 2176.5)	1639.6 (1289.9 to 2102.1)	1795.7 (1420.3 to 2283.7)	5.3 (-15.8 to 32.7)	5.6 (-15.6 to 35.1)	4.3 (-17.7 to 33.3)
	YLLs	Attributed all ages number	84320 (63607 to 109547)	41575 (31444 to 56181)	42745 (30861 to 56947)	206674 (159476 to 271523)	97894 (74434 to 131170)	108780 (82133 to 142627)	145.1 (88.3 to 227.9)	135.5 (76 to 221.2)	154.5 (94.3 to 248.2)
		Attributed age-standardized rate (per 100,000)	1513.4 (1160.9 to 1963.1)	1415.8 (1091.9 to 1833)	1628.1 (1216.5 to 2172.6)	1539.6 (1199.5 to 1991.9)	1438.2 (1109.1 to 1900.7)	1645.6 (1272.7 to 2115.3)	1.7 (-20.1 to 30.5)	1.6 (-20.1 to 32.6)	1.1 (-21.3 to 31.7)
	YLDs	Attributed all ages number	8039 (5829 to 10547)	4911 (3545 to 6439)	3128 (2224 to 4183)	32385 (23305 to 42874)	19520 (14001 to 26010)	12866 (9164 to 17092)	302.9 (268.9 to 349.8)	297.5 (253.5 to 365.4)	311.3 (270.3 to 353.7)
		Attributed age-standardized rate (per 100,000)	116.2 (85.7 to 152)	136.3 (99.8 to 179.4)	93.5 (66.3 to 123.8)	175.9 (129.4 to 232)	201.4 (146 to 270)	150.1 (108.6 to 202.8)	51.4 (40.3 to 66.7)	47.8 (32.7 to 71.2)	60.5 (46.2 to 75.4)

*Data in parentheses are 95% Uncertainty Intervals (95% UIs)

Supplementary Table 3. The causes trend of epidemiologic indices attributed to kidney dysfunction, attributed number for all ages and age-standardized rates, for males, females, and both sexes, in 1990 and 2019 and percent of changes in the 1990-2019 period.

Cause	Measure	Age, Metric	Year						% Change (1990 to 2019)		
			1990			2019					
			Both	Female	Male	Both	Female	Male	Both	Female	Male
Ischemic heart disease	Deaths	Attributed all ages number	54680 (39658 to 70920)	26789 (19328 to 34836)	27891 (19916 to 36668)	150471 (111836 to 191789)	69517 (50365 to 88829)	80954 (60333 to 103321)	175.2 (137.1 to 214.9)	16 (123.2 to 196)	190.3 (146.2 to 241.2)
		Attributed age-standardized rate (per 100,000)	40.6 (28.7 to 53.4)	40.2 (28.2 to 52.8)	40.5 (28.1 to 53.8)	43.7 (31.9 to 56.5)	41.4 (29.4 to 53.5)	45.8 (33.8 to 59)	7.7 (-6.6 to 22.3)	0.3 (-11 to 16.7)	13.2 (-3.4 to 32.3)
	DALYs	Attributed all ages number	1178338 (869221 to 1531091)	540128 (404489 to 686665)	638210 (460944 to 833258)	2942671 (2232367 to 3689021)	1278764 (962471 to 1608920)	1663907 (1269002 to 2092840)	149.7 (113.9 to 188.2)	13.7 (102.1 to 172.5)	160.7 (121.4 to 206)
		Attributed age-standardized rate (per 100,000)	733.5 (534.6 to 953)	693.4 (507.9 to 892.3)	768.2 (552.4 to 1010)	739.4 (556.9 to 932.3)	667.4 (497.2 to 841.2)	807.7 (606.1 to 1019.1)	0.8 (-13 to 16)	-0.4 (-17.6 to 10.2)	5.1 (-10.5 to 23.1)
	YLLs	Attributed all ages number	1161777 (858155 to 1506488)	533022 (398603 to 679555)	628755 (454238 to 821752)	2882286 (2190931 to 3618225)	1255018 (943974 to 1575810)	1627268 (1244310 to 2052641)	148.1 (111.9 to 187.1)	13.5 (100.4 to 171.4)	158.8 (119.4 to 204.4)
		Attributed age-standardized rate (per 100,000)	722.8 (526.5 to 937.9)	684.1 (500.8 to 881.6)	756 (540.8 to 990.6)	723.9 (545 to 911.7)	655.1 (486 to 828.2)	789.2 (594.8 to 998.6)	0.2 (-13.9 to 15.4)	-0.4 (-18.1 to 9.8)	4.4 (-11.2 to 22.3)
	YLDs	Attributed all ages number	16562 (10005 to 25915)	7106 (4332 to 10958)	9456 (5623 to 14816)	60385 (38012 to 92438)	23746 (15035 to 35832)	36639 (23082 to 56577)	264.6 (234.4 to 301)	23.4 (210.9 to 262.5)	287.5 (249.6 to 334.8)
		Attributed age-standardized rate (per 100,000)	10.8 (6.5 to 17)	9.3 (5.7 to 14.6)	12.2 (7.2 to 19.4)	15.5 (9.8 to 23.8)	12.4 (7.7 to 18.8)	18.5 (11.7 to 28.7)	43.4 (30.9 to 57.5)	3.3 (23.2 to 43.7)	51.1 (36.3 to 70)
Ischemic stroke	Deaths	Attributed all ages number	7702 (5480 to 10437)	4165 (2816 to 5798)	3537 (2551 to 4840)	24751 (16107 to 33178)	13002 (8257 to 17784)	11749 (7844 to 15757)	221.4 (154.1 to 281.9)	21.2 (148.9 to 282.6)	232.2 (154.8 to 302.2)
		Attributed age-standardized rate (per 100,000)	5.6 (3.5 to 7.9)	6 (3.6 to 8.6)	5.1 (3.4 to 7.2)	6.9 (4 to 9.7)	7.3 (4 to 10.5)	6.4 (3.9 to 9)	23.4 (-1.5 to 45.2)	2.1 (-4.1 to 47.8)	26.8 (-1.9 to 52.5)
	DALYs	Attributed all ages number	186725 (143799 to 237869)	102353 (76861 to 131586)	84372 (64944 to 109722)	601724 (459386 to 751793)	322260 (243957 to 409464)	279465 (212548 to 350218)	222.3 (172.6 to 270.6)	21.5 (167.9 to 269.6)	231.2 (167.3 to 291.6)
		Attributed age-standardized rate (per 100,000)	117.2 (85.5 to 153.6)	129.1 (92.3 to 171.4)	105 (77.5 to 140)	148.5 (107.9 to 190)	160.6 (114.7 to 209.1)	136.6 (99.3 to 176.3)	26.7 (6 to 45.3)	2.4 (5.7 to 46.1)	30.1 (5.9 to 53.3)

YLLs: Years of life lost, YLDs: Years lived with disability, DALYs: Disability-adjusted life years

The symbol −, used to denote that a number is negative and means decrease in % change

Cause	Measure	Age, Metric	Year						% Change (1990 to 2019)		
			1990			2019					
			Both	Female	Male	Both	Female	Male	Both	Female	Male
	YLLs	Attributed all ages number	155815 (119876 to 200477)	82562 (61604 to 108003)	73254 (55458 to 98306)	490283 (366299 to 623962)	253999 (186190 to 328376)	236284 (174496 to 298897)	214.7 (159.4 to 270.4)	20.8 (153.3 to 274.9)	222.6 (151.3 to 289.5)
		Attributed age-standardized rate (per 100,000)	99.4 (72.1 to 132.5)	106.1 (74.2 to 144.9)	92.3 (67.2 to 125.5)	122.8 (86.6 to 160.1)	128.9 (88.8 to 169.8)	116.9 (83.3 to 153)	23.6 (0.8 to 45)	2.1 (0.5 to 47.7)	26.7 (0.1 to 52.6)
	YLDs	Attributed all ages number	30910 (20617 to 41650)	19791 (13305 to 26585)	11118 (7412 to 14955)	111442 (75080 to 149264)	68260 (46013 to 91665)	43181 (29160 to 58292)	260.5 (241.1 to 279.2)	24.5 (227.3 to 262.4)	288.4 (260.4 to 313.5)
		Attributed age-standardized rate (per 100,000)	17.8 (11.9 to 24.3)	23 (15.3 to 31.4)	12.8 (8.4 to 17.5)	25.7 (17.2 to 35)	31.8 (21.3 to 43.4)	19.8 (13.1 to 27)	44 (35.7 to 52.3)	3.8 (30.8 to 45.7)	54.9 (43 to 66.2)
Intracerebral hemorrhage	Deaths	Attributed all ages number	5272 (4042 to 6780)	2914 (2154 to 3819)	2358 (1842 to 2999)	9316 (7042 to 11610)	4859 (3656 to 6118)	4458 (3385 to 5703)	76.7 (47.9 to 121.7)	6.7 (33.2 to 120.5)	89 (54.7 to 134.6)
		Attributed age-standardized rate (per 100,000)	3.5 (2.6 to 4.5)	3.8 (2.7 to 5.1)	3.1 (2.4 to 3.9)	2.4 (1.8 to 3.1)	2.6 (1.9 to 3.3)	2.3 (1.7 to 3)	-29.8 (-41.2 to -11.5)	-3.3 (-45.9 to -11.3)	-25.7 (-38.8 to -7.2)
	DALYs	Attributed all ages number	139048 (108956 to 176169)	76672 (57890 to 99012)	62375 (48921 to 79112)	233831 (183702 to 289765)	121413 (94803 to 150377)	112418 (86731 to 143786)	68.2 (41.3 to 109.5)	5.8 (28.2 to 105.5)	80.2 (48.2 to 121.5)
		Attributed age-standardized rate (per 100,000)	77.4 (60.5 to 98.9)	86.3 (64.7 to 112.2)	68.5 (53.9 to 86.7)	52.3 (41 to 64.9)	55.6 (43 to 69)	49.3 (38.1 to 62)	-32.4 (-42.9 to -15.8)	-3.6 (-47.7 to -15.9)	-28.1 (-40.6 to -11.6)
	YLLs	Attributed all ages number	132827 (103271 to 169647)	72821 (54127 to 94415)	60007 (46805 to 76430)	218877 (171825 to 272047)	112705 (87626 to 140393)	106172 (81446 to 136967)	64.8 (37 to 107.6)	5.5 (23.6 to 104)	76.9 (44.4 to 119.6)
		Attributed age-standardized rate (per 100,000)	74.2 (57.5 to 95.2)	82.3 (60.7 to 107.2)	66.1 (51.9 to 84.1)	49.2 (38.4 to 61)	51.9 (40 to 64.8)	46.7 (35.9 to 59.3)	-33.6 (-44.4 to -16.7)	-3.7 (-49.5 to -16.5)	-29.3 (-42 to -12.4)
	YLDs	Attributed all ages number	6220 (4094 to 8586)	3851 (2540 to 5365)	2369 (1560 to 3258)	14954 (9997 to 20813)	8708 (5878 to 11962)	6246 (4192 to 8711)	140.4 (125.3 to 157.3)	12.6 (111.1 to 143.1)	163.7 (144.6 to 188.2)
		Attributed age-standardized rate (per 100,000)	3.2 (2.1 to 4.4)	4 (2.7 to 5.5)	2.4 (1.6 to 3.3)	3.1 (2.1 to 4.3)	3.7 (2.5 to 5.1)	2.5 (1.7 to 3.5)	-2.5 (-8.6 to 4.2)	-0.7 (-13.6 to -0.2)	6.2 (-1.9 to 15.7)

Cause	Measure	Age, Metric	Year						% Change (1990 to 2019)		
			1990			2019					
			Both	Female	Male	Both	Female	Male	Both	Female	Male
Peripheral artery disease	Deaths	Attributed all ages number	62 (43 to 88)	21 (12 to 39)	42 (29 to 59)	281 (187 to 394)	103 (56 to 173)	178 (126 to 233)	351 (216.5 to 496.6)	39.7 (200.5 to 672.5)	328.2 (175.8 to 504.9)
		Attributed age-standardized rate (per 100,000)	0 (0 to 0.1)	0 (0 to 0.1)	0.1 (0 to 0.1)	0.1 (0 to 0.1)	0.1 (0 to 0.1)	0.1 (0.1 to 0.1)	85.4 (30.2 to 144.9)	10.2 (24.3 to 222.1)	76.6 (15.7 to 145.9)
	DALYs	Attributed all ages number	3093 (2053 to 4598)	1404 (832 to 2286)	1690 (1148 to 2412)	10697 (7277 to 15431)	4588 (2867 to 7323)	6108 (4346 to 8398)	245.8 (184.3 to 313.2)	22.7 (173.2 to 291.9)	261.5 (173.2 to 359.6)
		Attributed age-standardized rate (per 100,000)	1.9 (1.2 to 2.9)	1.8 (1 to 3)	2 (1.3 to 2.8)	2.7 (1.8 to 3.9)	2.4 (1.5 to 3.8)	2.9 (2 to 4)	41.3 (18.6 to 66.6)	3.4 (12.2 to 61)	48 (12.5 to 84.6)
	YLLs	Attributed all ages number	1490 (1068 to 2038)	432 (270 to 708)	1058 (719 to 1547)	5845 (4391 to 7633)	1834 (1149 to 2725)	4011 (3024 to 5333)	292.4 (173.2 to 428.3)	32.4 (158.6 to 537.7)	279.3 (146.2 to 447.7)
		Attributed age-standardized rate (per 100,000)	0.9 (0.6 to 1.2)	0.5 (0.3 to 0.9)	1.2 (0.8 to 1.7)	1.4 (1 to 1.9)	1 (0.6 to 1.5)	1.8 (1.4 to 2.4)	63 (15.2 to 115.7)	7.8 (7.5 to 172.6)	56.6 (3.9 to 120.7)
	YLDs	Attributed all ages number	1604 (725 to 2945)	972 (447 to 1802)	632 (284 to 1175)	4852 (2237 to 8943)	2755 (1280 to 5108)	2098 (952 to 3918)	202.6 (184.2 to 222.3)	18.4 (165.7 to 202.1)	231.9 (205.9 to 258)
		Attributed age-standardized rate (per 100,000)	1 (0.5 to 1.9)	1.2 (0.6 to 2.3)	0.8 (0.4 to 1.5)	1.2 (0.6 to 2.3)	1.4 (0.6 to 2.6)	1.1 (0.5 to 2)	22.7 (15.8 to 30.3)	1.5 (8.1 to 22.6)	35.1 (25.3 to 45.6)
Gout	YLDs	Attributed all ages number	3205 (1932 to 4722)	944 (575 to 1392)	2261 (1368 to 3319)	14550 (8891 to 21349)	3795 (2360 to 5549)	10755 (6543 to 15920)	354 (331.7 to 377.9)	30.2 (271.2 to 334.7)	375.7 (349.3 to 404.6)
		Attributed age-standardized rate (per 100,000)	2.1 (1.3 to 3.1)	1.3 (0.8 to 1.9)	3.1 (1.9 to 4.5)	3.7 (2.3 to 5.5)	2 (1.2 to 2.9)	5.5 (3.3 to 8)	74.1 (65.5 to 83.1)	5.6 (44 to 68.4)	78.7 (68.1 to 89.5)
Chronic kidney disease due to diabetes mellitus type 1	Deaths	Attributed all ages number	2211 (1427 to 3179)	1011 (648 to 1467)	1200 (773 to 1746)	4663 (2917 to 7071)	2091 (1296 to 3130)	2572 (1568 to 3983)	110.9 (75.4 to 154.3)	10.7 (60.4 to 151.3)	114.4 (72.2 to 174.6)
		Attributed age-standardized rate (per 100,000)	1.1 (0.7 to 1.6)	1 (0.6 to 1.4)	1.2 (0.7 to 1.8)	0.9 (0.6 to 1.4)	0.8 (0.5 to 1.2)	1 (0.6 to 1.5)	-17 (-31.7 to 0.3)	-1.6 (-35.4 to 2)	-18.3 (-35.3 to 5.3)

Cause	Measure	Age, Metric	Year						% Change (1990 to 2019)		
			1990			2019					
			Both	Female	Male	Both	Female	Male	Both	Female	Male
	DALYs	Attributed all ages number	90807 (61823 to 127057)	43144 (29318 to 60581)	47662 (31896 to 67554)	196520 (130991 to 285106)	89570 (59440 to 129439)	106949 (69635 to 158458)	116.4 (83 to 157.2)	10.8 (66.3 to 150.6)	124.4 (86.8 to 178.5)
		Attributed age-standardized rate (per 100,000)	39.3 (25.9 to 55.6)	37.1 (24.5 to 53.1)	41.5 (27 to 59.7)	34 (22.3 to 49.5)	32 (20.8 to 46.8)	35.9 (23.2 to 53.4)	-13.5 (-27 to 2.3)	-1.4 (-31 to 3.5)	-13.3 (-28.4 to 8.2)
	YLLs	Attributed all ages number	84098 (55594 to 119376)	40051 (26406 to 57355)	44047 (28372 to 63530)	172507 (110340 to 259217)	78869 (49454 to 117722)	93638 (58132 to 144739)	105.1 (70.5 to 149.1)	9.7 (53.5 to 142.4)	112.6 (72.5 to 172.9)
		Attributed age-standardized rate (per 100,000)	37 (23.8 to 53.2)	35 (22.7 to 50.6)	38.9 (24.9 to 56.9)	30 (18.9 to 45.1)	28.4 (17.5 to 42.3)	31.6 (19.4 to 48.8)	-18.8 (-32.3 to -2)	-1.9 (-37 to -1.3)	-18.8 (-34.4 to 4)
	YLDs	Attributed all ages number	6708 (4468 to 9229)	3093 (2089 to 4329)	3615 (2364 to 4956)	24013 (15623 to 33779)	10701 (7039 to 15243)	13311 (8599 to 18362)	258 (217.9 to 302.1)	24.6 (202 to 293.2)	268.2 (228 to 315.7)
		Attributed age-standardized rate (per 100,000)	2.3 (1.6 to 3.2)	2.1 (1.5 to 3)	2.5 (1.7 to 3.5)	4 (2.6 to 5.6)	3.6 (2.4 to 5.1)	4.3 (2.8 to 6)	69.5 (53.3 to 86.8)	6.9 (49.8 to 89.9)	70.1 (53.5 to 88.5)
Chronic kidney disease due to diabetes mellitus type 2	Deaths	Attributed all ages number	15138 (11845 to 19222)	7378 (5708 to 10042)	7759 (5937 to 10155)	34939 (26926 to 43879)	17500 (13130 to 22193)	17440 (13413 to 22803)	130.8 (84.3 to 177.8)	13.7 (75.4 to 183.7)	124.8 (73.2 to 192.8)
		Attributed age-standardized rate (per 100,000)	10.7 (8.3 to 14)	10.2 (7.8 to 14.4)	11.3 (8.7 to 15.4)	9.6 (7.5 to 12)	9.6 (7.3 to 12.2)	9.6 (7.4 to 12.4)	-10.2 (-28.9 to 8.2)	-0.6 (-29.8 to 12)	-15.2 (-35.1 to 10.7)
	DALYs	Attributed all ages number	370212 (293306 to 453591)	182624 (143823 to 231258)	187588 (145657 to 235316)	863703 (678142 to 1069675)	429142 (327345 to 534691)	434561 (337706 to 566561)	133.3 (90.5 to 175.7)	13.5 (80.3 to 178.1)	131.7 (82.3 to 193.4)
		Attributed age-standardized rate (per 100,000)	222.2 (175.5 to 274.4)	218.8 (172 to 285.7)	226.5 (177.2 to 287.6)	205.9 (162.4 to 253.6)	207.4 (160.2 to 257)	204.8 (159.8 to 263.7)	-7.3 (-24.6 to 9.3)	-0.5 (-27.8 to 11.7)	-9.6 (-29.6 to 14.3)
	YLLs	Attributed all ages number	337829 (262956 to 417823)	165109 (128220 to 211693)	172721 (132800 to 219189)	736967 (556415 to 939888)	368684 (272990 to 468290)	368283 (276841 to 494603)	118.1 (74.4 to 163.5)	12.3 (66.1 to 169)	113.2 (64.7 to 177.2)
		Attributed age-standardized rate (per 100,000)	203.5 (159.4 to 254.8)	198.6 (154.1 to 262.2)	209.3 (161.9 to 270.7)	176.6 (133.7 to 223.8)	179.2 (133.8 to 227.9)	174.1 (133 to 230.8)	-13.2 (-31.2 to 4.6)	-1 (-33.2 to 8.3)	-16.8 (-36.1 to 8.8)

Cause	Measure	Age, Metric	Year						% Change (1990 to 2019)		
			1990			2019					
			Both	Female	Male	Both	Female	Male	Both	Female	Male
	YLDs	Attributed all ages number	32382 (23111 to 43716)	17515 (12538 to 23683)	14867 (10452 to 20598)	126735 (88802 to 174788)	60458 (42714 to 81417)	66278 (45334 to 93315)	291.4 (267.9 to 315.8)	24.5 (221.9 to 269.9)	345.8 (315.5 to 377.7)
		Attributed age-standardized rate (per 100,000)	18.6 (13.5 to 25.6)	20.1 (14.5 to 27.3)	17.2 (12.1 to 24)	29.4 (20.7 to 41)	28.1 (20 to 38.5)	30.7 (20.9 to 44.1)	57.6 (47.9 to 67.8)	4 (30.8 to 49.5)	78.6 (66.2 to 91.2)
Chronic kidney disease due to glomerulonephritis	Deaths	Attributed all ages number	3842 (2997 to 4946)	1734 (1316 to 2257)	2108 (1626 to 2763)	7094 (5189 to 9513)	3056 (2171 to 4095)	4038 (2954 to 5534)	84.6 (52.3 to 121.7)	7.6 (38.3 to 114.7)	91.5 (57.9 to 138.8)
		Attributed age-standardized rate (per 100,000)	2 (1.5 to 2.7)	1.7 (1.3 to 2.3)	2.4 (1.7 to 3.3)	1.7 (1.2 to 2.3)	1.5 (1 to 2)	1.9 (1.4 to 2.7)	-16.9 (-31.1 to -1.3)	-1.5 (-33.7 to 0.6)	-18.9 (-35.4 to 2.5)
	DALYs	Attributed all ages number	195850 (155316 to 245221)	97168 (74423 to 124413)	98682 (77000 to 123890)	346681 (271049 to 432787)	160370 (124447 to 201430)	186311 (145850 to 237698)	77 (50.1 to 108.9)	6.5 (31.6 to 98.1)	88.8 (58.7 to 128.7)
		Attributed age-standardized rate (per 100,000)	71.2 (57.6 to 87.3)	67.8 (54 to 84.4)	74.7 (60.1 to 93.5)	64 (50.2 to 79.8)	60 (46.3 to 74.8)	67.9 (53.5 to 86.6)	-10.1 (-21.7 to 2.9)	-1.2 (-25.6 to 2.6)	-9.2 (-21.5 to 7.3)
	YLLs	Attributed all ages number	154012 (115301 to 198327)	75147 (54754 to 101422)	78865 (59054 to 103087)	221112 (162600 to 293928)	98220 (69731 to 133117)	122892 (89265 to 166965)	43.6 (14.5 to 80.3)	3.1 (-3.4 to 67.7)	55.8 (24.3 to 101.8)
		Attributed age-standardized rate (per 100,000)	57.8 (44.7 to 73.1)	53.6 (40.5 to 69.5)	62.2 (48.1 to 80.7)	43.3 (32.4 to 56.6)	39.1 (28.1 to 52.6)	47.5 (34.7 to 64.9)	-25 (-37 to -10.7)	-2.7 (-42 to -11.5)	-23.7 (-36.3 to -5.8)
	YLDs	Attributed all ages number	41838 (28339 to 57326)	22021 (14807 to 30085)	19817 (13391 to 27249)	125569 (84364 to 171768)	62150 (42324 to 85778)	63419 (42908 to 87518)	200.1 (174.3 to 230.5)	18.2 (155.6 to 212)	220 (189 to 256.5)
		Attributed age-standardized rate (per 100,000)	13.4 (9.1 to 18.3)	14.2 (9.6 to 19.5)	12.6 (8.6 to 17.2)	20.6 (14 to 28.2)	20.9 (14.3 to 28.7)	20.4 (13.8 to 28)	54.1 (46.5 to 63)	4.7 (37.2 to 57.6)	62.3 (52.1 to 73.6)
Chronic kidney disease due to hypertension	Deaths	Attributed all ages number	15619 (12632 to 20398)	7177 (5644 to 9941)	8442 (6710 to 11202)	36295 (29062 to 44169)	17162 (13513 to 21437)	19134 (15244 to 24154)	132.4 (87.7 to 178)	13.9 (77.1 to 188.8)	126.6 (76.3 to 193.4)
		Attributed age-standardized rate (per 100,000)	11.9 (9.6 to 15.9)	10.6 (8.4 to 15.3)	13.5 (10.6 to 18.3)	10.6 (8.5 to 12.8)	10 (7.9 to 12.4)	11.2 (9 to 14.1)	-11.3 (-29.5 to 6.3)	-0.6 (-32.3 to 13)	-17 (-36.6 to 6.8)

Cause	Measure	Age, Metric	Year						% Change (1990 to 2019)		
			1990			2019					
			Both	Female	Male	Both	Female	Male	Both	Female	Male
	DALYs	Attributed all ages number	360774 (295600 to 451184)	166952 (134223 to 216321)	193822 (157373 to 242611)	834868 (674719 to 1006266)	390170 (312204 to 487434)	444699 (356201 to 556028)	131.4 (92.9 to 172.2)	13.4 (81.4 to 178.8)	129.4 (85.1 to 187.8)
		Attributed age-standardized rate (per 100,000)	222.4 (183.4 to 282.6)	203.6 (163.5 to 271.3)	242.6 (197.8 to 311.6)	203.3 (165.8 to 243)	192.3 (154.2 to 238.2)	214.1 (173.9 to 265.3)	-8.6 (-24.8 to 7.6)	-0.6 (-27.9 to 12.4)	-11.7 (-30.7 to 11)
	YLLs	Attributed all ages number	325456 (261340 to 413515)	148943 (116526 to 197979)	176513 (140725 to 226934)	699205 (552248 to 873117)	328683 (253487 to 422589)	370522 (287215 to 478198)	114.8 (75.5 to 158.3)	12.1 (66.5 to 170.1)	109.9 (64.4 to 172.1)
		Attributed age-standardized rate (per 100,000)	204.6 (166.6 to 264.8)	185.7 (146.3 to 252.8)	224.9 (179.6 to 296.2)	174.6 (139 to 215.2)	166.4 (130.4 to 210.1)	182.6 (144.3 to 233.2)	-14.7 (-31 to 2.5)	-1 (-33.2 to 8.6)	-18.8 (-37.4 to 4.8)
	YLDs	Attributed all ages number	35318 (24784 to 46931)	18009 (12691 to 23986)	17309 (11963 to 23211)	135663 (95488 to 181018)	61487 (43629 to 81233)	74177 (51527 to 100528)	284.1 (261.7 to 307.8)	24.1 (218.9 to 265.5)	328.6 (297.9 to 358.9)
		Attributed age-standardized rate (per 100,000)	17.8 (12.9 to 23.7)	17.9 (13.1 to 23.9)	17.7 (12.3 to 23.9)	28.7 (20.6 to 38.7)	25.9 (18.6 to 34.2)	31.6 (21.8 to 43.6)	61.4 (53.2 to 70)	4.5 (36.4 to 53.3)	78.1 (66.2 to 89.6)
Chronic kidney disease due to other and unspecified causes	Deaths	Attributed all ages number	15786 (12891 to 19502)	8893 (7180 to 10963)	6893 (5511 to 8807)	28820 (21720 to 37145)	16591 (12403 to 21351)	12230 (8918 to 16906)	82.6 (55.6 to 113.2)	8.7 (51.9 to 120.2)	77.4 (49 to 120.9)
		Attributed age-standardized rate (per 100,000)	9 (6.9 to 11.9)	10.1 (7.7 to 13.7)	7.8 (5.7 to 11)	7.6 (5.6 to 9.8)	8.9 (6.7 to 11.4)	6.3 (4.5 to 8.9)	-15.7 (-30.9 to -1.3)	-1.2 (-31.9 to 3.9)	-20.2 (-35.7 to 1.5)
	DALYs	Attributed all ages number	749397 (618148 to 877438)	427544 (352240 to 509633)	321853 (249198 to 389664)	1139551 (925136 to 1381090)	650116 (524845 to 789151)	489436 (386730 to 620268)	52.1 (30.2 to 81)	5.2 (23.8 to 80.9)	52.1 (23.7 to 95.1)
		Attributed age-standardized rate (per 100,000)	279.9 (236.7 to 333)	326.3 (273.8 to 388)	233.9 (193.7 to 286.5)	237.2 (191.1 to 288.4)	278.4 (223.6 to 336.4)	197.6 (156.3 to 252.1)	-15.3 (-24.7 to -5)	-1.5 (-26.7 to -2.7)	-15.5 (-26.4 to -0.4)
	YLLs	Attributed all ages number	629487 (494702 to 748384)	348088 (274832 to 427089)	281399 (209952 to 345938)	786546 (611988 to 1004505)	433194 (327447 to 553639)	353352 (264425 to 475670)	25 (2.3 to 54.3)	2.4 (-5.5 to 54.5)	25.6 (-1.3 to 71.3)
		Attributed age-standardized rate (per 100,000)	228.6 (188.5 to 278.2)	257.6 (210.4 to 315.2)	199.8 (160 to 250.6)	165.8 (128.6 to 212.1)	189.6 (142.6 to 241.2)	142.7 (106.5 to 192.7)	-27.4 (-38.1 to -15.2)	-2.6 (-40.6 to -12.2)	-28.6 (-39.7 to -11.9)

Cause	Measure	Age, Metric	Year						% Change (1990 to 2019)		
			1990			2019					
			Both	Female	Male	Both	Female	Male	Both	Female	Male
	YLDs	Attributed all ages number	119910 (87709 to 156634)	79456 (58106 to 104712)	40454 (29209 to 53471)	353005 (260120 to 456221)	216922 (159798 to 280928)	136084 (98071 to 180063)	194.4 (175.6 to 216.4)	17.3 (155.7 to 191.6)	236.4 (207.8 to 266.4)
		Attributed age-standardized rate (per 100,000)	51.3 (37.9 to 66.8)	68.7 (50.7 to 90.7)	34.1 (25.1 to 44.5)	71.4 (52.9 to 92.2)	88.7 (66.1 to 114.9)	54.9 (40.1 to 72.7)	39 (31.3 to 47.1)	2.9 (22.1 to 36.9)	61.3 (49.9 to 73.2)

*Data in parentheses are 95% Uncertainty Intervals (95% UIs)

Supplementary Figure 1. Rates of YLLs and YLDs attributable to kidney dysfunction in categories of age-standardized, under 20, 20 to 54 years, and 55 plus in 21 countries in years 1990 and 2019.

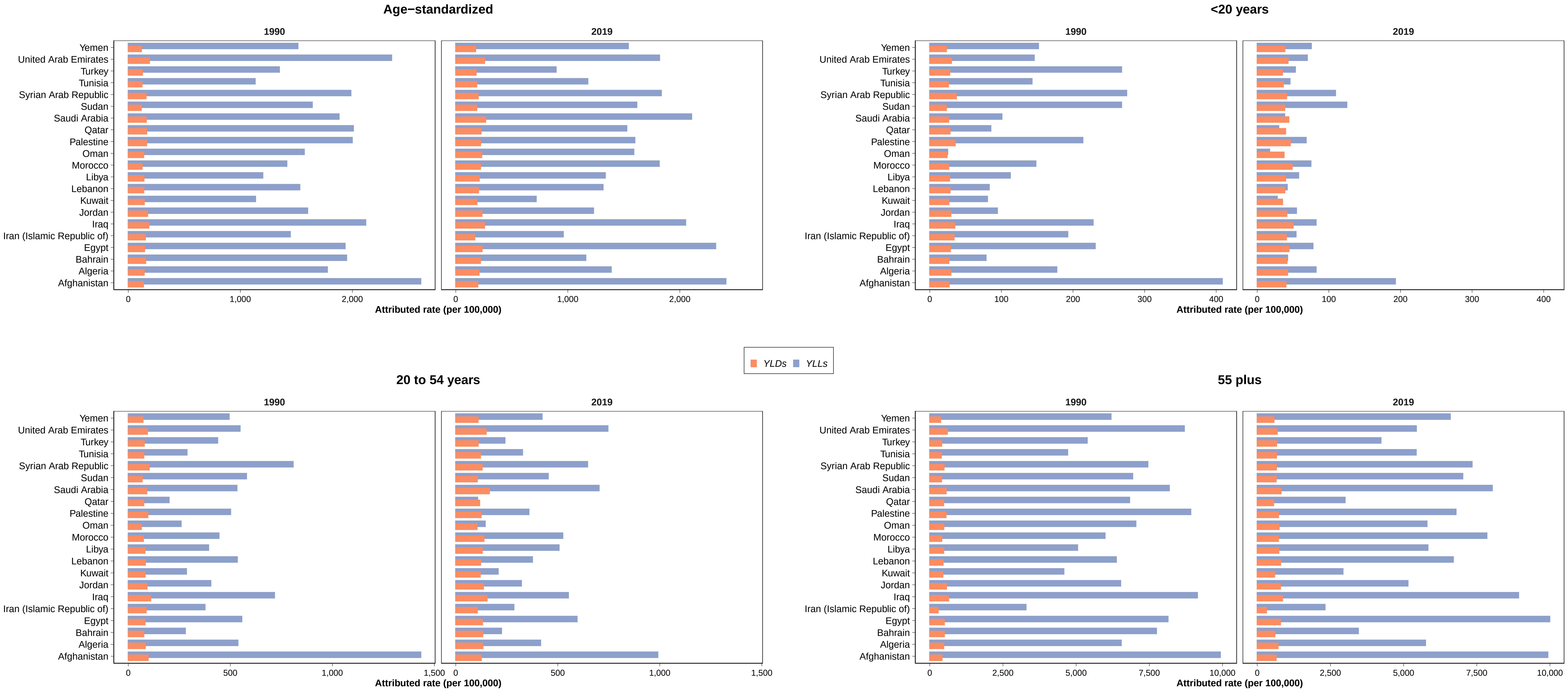

YLLs: Years of life lost, YLDs: Years lived with disability

Supplementary Figure 2. Attributed age-standardized rate of YLLs, YLDs, Deaths, and DALYs of kidney dysfunction of each country of the region in both sexes by SDI quintiles from 1990 to 2019.

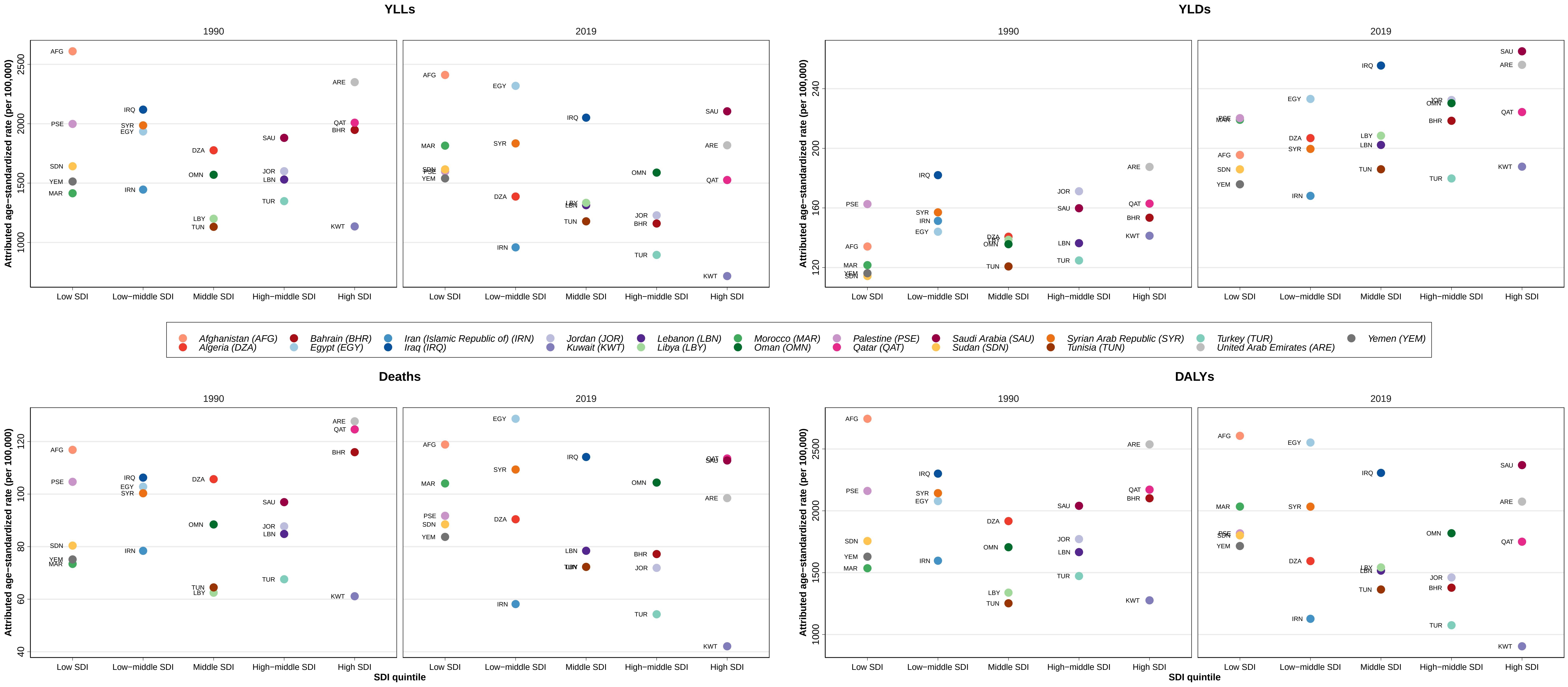

YLLs: Years of life lost, YLDs: Years lived with disability, DALYs: Disability-adjusted life years, SDI: Socio-demographic index

Authors’ Contributions

Providing data or critical feedback on data sources

Ayman Ahmed, Jalal Arabloo, Seyyed Shamsadin Athari, Sina Azadnajafabad, Sara Bagherieh, Luciana Aparecida Campos, Farshad Farzadfar, Ali Fatehizadeh, Hadi Hassankhani, Mahsa Heidari-Foroozan, Kamran Hessami, Rana Irilouzadian, Haitham Jahrami, Sathish Kumar Jayapal, Moien AB Khan, Mohammed Kuddus, Ata Mahmoodpoor, Mohammadreza Mobayen, Esmaeil Mohammadi, Faezeh Mohammadi, Syam Mohan, Ali H Mokdad, Sara Momtazmanesh, Christopher J L Murray, Mohsen Naghavi, Zuhair S Natto, Amirfarzan Rashidi, Elrashdy Moustafa Mohamed Redwan, Aly M A Saad, Sahar Saeedi Moghaddam, Abdallah M Samy, Jeevan K Shetty, Mohammad Tabish, Amir Taherkhani, and Muhammad Umair.

Developing methods or computational machinery

Sina Azadnajafabad, Zahra Esfahani, Farshad Farzadfar, Ali H Mokdad, Christopher J L Murray, Mohsen Naghavi, Mohammad-Mahdi Rashidi, and Sahar Saeedi Moghaddam.

Providing critical feedback on methods or results

Samar Abd ElHafeez, Sherief Abd-Elsalam, Meriem Abdoun, Aqeel Ahmad, Ayman Ahmed, Rajaa M Al-Raddadi, Ala'a B Al-Tammemi, Javad Aminian Dehkordi, Mehrdad Amir-Behghadami, Jalal Arabloo, Mohammad Athar, Seyyed Shamsadin Athari, Sina Azadnajafabad, Mahsa Babaei, Hassan Babamohamadi, Nayereh Baghcheghi, Sara Bagherieh, Akshaya Srikanth Bhagavathula, Vijayalakshmi S Bhojaraja, Milad Bonakdar Hashemi, Luciana Aparecida Campos, Azizallah Dehghan, Muhammed Elhadi, Waseem El-Huneidi, Farshad Farzadfar, Ali Fatehizadeh, Alireza Feizkhah, Ali Gholami, Samer Hamidi, Hadi Hassankhani, Kamran Hessami, Kamal Hezam, Mohammad-Salar Hosseini, Soodabeh Hoveidamanesh, Rana Irilouzadian, Haitham Jahrami, Tannaz Jamialahmadi, Sathish Kumar Jayapal, Mohammad Keykhaei, Amirmohammad

Khalaji, Moien AB Khan, Yusra H Khan, Moawiah Mohammad Khatatbeh, Farzad Kompani, Hamid Reza Koohestani, Mohammed Kuddus, Bagher Larijani, Ata Mahmoodpoor, Elaheh Malakan Rad, Mohammad-Reza Malekpour, Ahmad Azam Malik, Tauqeer Hussain Mallhi, Mohammadreza Mobayen, Esmaeil Mohammadi, Faezeh Mohammadi, Syam Mohan, Ali H Mokdad, Sara Momtazmanesh, Christopher J L Murray, Mohsen Naghavi, Zuhair S Natto, Seyed Aria Nejadghaderi, Hassan Okati-Aliabad, Sina Rashedi, Mahsa Rashidi, Mohammad-Mahdi Rashidi, Elrashdy Moustafa Mohamed Redwan, Nazila Rezaei, Negar Rezaei, Aly M A Saad, Sahar Saeedi Moghaddam, Fatemeh Saheb Sharif-Askari, Morteza Saki, Abdallah M Samy, Elaheh Shaker, Jeevan K Shetty, Seyed Afshin Shorofi, Mohammad Tabish, Muhammad Umair, Seyed Mohammad Vahabi, Fereshteh Yazdanpanah, Arzu Yigit, Mazyar Zahir, and Moein Zangiabadian.

Drafting the work or revising it critically for important intellectual content

Mohsen Abbasi-Kangevari, Zeinab Abbasi-Kangevari, Samar Abd ElHafeez, Sherief Abd-Elsalam, Eman Abu-Gharbieh, Ayman Ahmed, Sayer Al-Azzam, Ala'a B Al-Tammemi, Mehrdad Amir-Behghadami, Jalal Arabloo, Seyyed Shamsadin Athari, Sina Azadnajafabad, Mahsa Babaei, Sara Bagherieh, Hamid Reza Baradaran, Akshaya Srikanth Bhagavathula, Vijayalakshmi S Bhojaraja, Milad Bonakdar Hashemi, Luciana Aparecida Campos, Muhammed Elhadi, Farshad Farzadfar, Ali Fatehizadeh, Alireza Feizkhah, Fataneh Ghadirian, Kamran Hessami, Kamal Hezam, Mohammad-Salar Hosseini, Rana Irilouzadian, Haitham Jahrami, Tannaz Jamialahmadi, Sathish Kumar Jayapal, Reema A Karasneh, Amirmohammad Khalaji, Moien AB Khan, Yusra H Khan, Moawiah Mohammad Khatatbeh, Farzad Kompani, Mohammed Kuddus, Bagher Larijani, Soleiman Mahjoub, Ata Mahmoodpoor, Elaheh Malakan Rad, Mohammad-Reza Malekpour, Ahmad Azam Malik, Tauqeer Hussain Mallhi, Esmaeil Mohammadi, Faezeh Mohammadi, Ali H Mokdad, Sara Momtazmanesh, Christopher J L Murray, Mohsen Naghavi, Zuhair S Natto, Seyed Aria Nejadghaderi, Sina Rashedi, Mohammad-Mahdi Rashidi, Nazila Rezaei, Negar Rezaei, Aly M A Saad, Sahar Saeedi Moghaddam, Fatemeh Saheb Sharif-Askari, Amirhossein Sahebkar, Abdallah M Samy, Elaheh Shaker, Jeevan K Shetty, Seyed Afshin Shorofi, Mohammad Tabish, Muhammad Umair, Arzu Yigit, and Mazyar Zahir.

Managing the estimation or publications process

Sina Azadnajafabad, Bagher Larijani, Ali H Mokdad, Christopher J L Murray, Mohsen Naghavi, Mohammad-Mahdi Rashidi, Nazila Rezaei, Negar Rezaei, and Sahar Saeedi Moghaddam.
